# Diversity and Taxonomic Distribution of Endophytic Bacterial Community in the Rice Plant and Its Prospective

**DOI:** 10.3390/ijms221810165

**Published:** 2021-09-21

**Authors:** Mohsin Ali, Qurban Ali, Muhammad Aamir Sohail, Muhammad Furqan Ashraf, Muhammad Hamzah Saleem, Saddam Hussain, Lei Zhou

**Affiliations:** 1 State Key Laboratory for Managing Biotic and Chemical Threats to the Quality and Safety of Agro-Products, Institute of Agro-Product Safety and Nutrition, Zhejiang Academy of Agricultural Sciences, Hangzhou 310021, China; moh.uaf2356@outlook.com; 2 State Key Laboratory of Microbial Resources, Institute of Microbiology, Chinese Academy of Sciences, Beijing 100101, China; 3 Key Laboratory of Integrated Management of Crop Diseases and Pests, Ministry of Education, Department of Plant Pathology, College of Plant Protection, Nanjing Agricultural University, Nanjing 210095, China; 4 Center for Excellence in Molecular Plant Sciences, National Key Laboratory of Plant Molecular Genetics, Institute of Plant Physiology and Ecology, Chinese Academy of Sciences, 300 Fenglin Road, Shanghai 200032, China; amirsohail306@gmail.com; 5 College of Life Sciences, South China Agricultural University, Guangzhou 510642, China; furqan2210uaf@hotmail.com; 6 College of Plant Science and Technology, Huazhong Agricultural University, Wuhan 430070, China; saleemhamza312@webmail.hzau.edu.cn; 7 Department of Agronomy, University of Agriculture, Faisalabad 38040, Punjab, Pakistan; sadamhussainuaf@gmail.com

**Keywords:** rice, plant–microbe interaction, endophytic bacteria, taxonomic afflictions, plant-growth promotion (PGP) traits, biocontrol

## Abstract

Endophytic bacterial communities are beneficial communities for host plants that exist inside the surfaces of plant tissues, and their application improves plant growth. They benefit directly from the host plant by enhancing the nutrient amount of the plant’s intake and influencing the phytohormones, which are responsible for growth promotion and stress. Endophytic bacteria play an important role in plant-growth promotion (PGP) by regulating the indirect mechanism targeting pest and pathogens through hydrolytic enzymes, antibiotics, biocontrol potential, and nutrient restriction for pathogens. To attain these benefits, firstly bacterial communities must be colonized by plant tissues. The nature of colonization can be achieved by using a set of traits, including attachment behavior and motility speed, degradation of plant polymers, and plant defense evasion. The diversity of bacterial endophytes colonization depends on various factors, such as plants’ relationship with environmental factors. Generally, each endophytic bacteria has a wide host range, and they are used as bio-inoculants in the form of synthetic applications for sustainable agriculture systems and to protect the environment from chemical hazards. This review discusses and explores the taxonomic distribution of endophytic bacteria associated with different genotypes of rice plants and their origin, movement, and mechanism of PGP. In addition, this review accentuates compressive meta data of endophytic bacteria communities associated with different genotypes of rice plants, retrieves their plant-growth-promoting properties and their antagonism against plant pathogens, and discusses the indication of endophytic bacterial flora in rice plant tissues using various methods. The future direction deepens the study of novel endophytic bacterial communities and their identification from rice plants through innovative techniques and their application for sustainable agriculture systems.

## 1. Introduction

Plants can build a relationship with their ecosystem members to enhance growth and development in natural environments, as well as maintaining an ecological niche for thriving microbes. Numerous kinds of microorganisms, including bacterial and fungal communities, are harboring plant compartments that have been isolated and identified by using plant tissues and are nominated as endophytes. A majority of endophytes colonize with different parts of the plant apoplast, proliferating among the cellular spaces, including xylem vessels. Endophytes can also colonize plant reproductive organs, such as flower buds, flower petals, fruits, and seeds [1]. For the first time, the isolation of the endophytic bacteria was carried out by Samish and Mund [2] after surface sterilization of the plant tissues. Afterward, many researchers successfully categorized and reported more than 200 genera of endophytic bacteria from different plant tissues. These genera belong to the 16 phyla that are culturable and unculturable endophytes: Acidobacteria, Aquificae, Actinobacteria, Bacteroidetes, Chloroflexi, Chlorobi, Cyanobacteria, Proteobacteria, Firmicutes, Deinococcus-thermus, Fusobacteria, Planctomycetes, Gemmatimonates, Nitrospira, Spirochaetes, and Verrucomicrobia [1,3]. However, there is no such compressive report on phylum numbers identified from rice plant tissues. The previous literature revealed that several endophytes represent the three main phyla, namely Proteobacteria, Firmicutes, and Actinobacteria, comprising members of *Azoarcus* [4], *Bacillus* [5], *Enterobacter* [6], *Gluconobacter*, *Stentrophomonas* [7], *Herbaspirillum* [8], *Pseudomonas*, *Serratia* [9], and *Streptomyces* [10].

Rice (*Oryza sativa* L.) is the most widely important grown cereal crop and is consumed by more than 50% of the world’s population [11]. The ever-increasing world population demands sustainable agriculture production to feed 7.3 billion people; that population may become 9.7 billion up to 2064 (estimated by the United Nations (UN) [12,13]). An increasing population needs a high yield of rice to compete for this competition, but it must be achieved without, or by minimizing, the application of synthetic products due to high concern about environmental protection. Still, the main source of crop improvement in the agriculture sector is the practice of applying synthetic products, such as commercial fertilizers, nutrient supplements, insecticides, and pesticides. This action has hazardous effects on the environment and human and livestock health. The farmer communities are convinced to change their old practices by using alternative products that could be environmentally friendly [14]. The bio-fertilizers and pesticides consist of non-pathogenic microorganisms; this uniqueness in nature facilitates attaining sustainable agriculture and environmental protection from chemical hazards. Additionally, bio-formulations might be the best alternative option to reduce environmental degradation and the threat to human health.

In the European Union Pesticides Database [15], several registered bio-fertilizers and pesticides containing bacterial products are mainly synthesized by using *Bacillus* species and *Pseudomonas* species. In China, Fang [16] reported the sources of registered bio-fertilizer products; these can be the single and combination of the bacterial strains of *Bacillus* species (*B. licheniformis*, *B. amyloliguefaciens*, *B. megaterium*, and *B. subtilis*) and *Lactobacillus plantarum*, *Paemibacillus mucilaginosus*, and other species. These abovementioned members of genera are dominated among plants, soil, and under other environments, due to salient features, such as rapid proliferation and simple diet requirements, which can assist in easy propagation of bacteria in the environment. The above-highlighted bacteria are famous as beneficial organisms for plant growth, as well as for producing many metabolites to improve plant health. Therefore, these genera could be the best choice for preparing potential inoculants by mixing bacterial strains due to their diversified adaption to multiple environmental factors, such as pH, low and high temperature, salinity, and high metals, as well as the ability of their cells to remain alive in diverse environments for a long time [17]. Importantly, the endophytic bacterial strains that are beneficial for growth promotion and disease suppression will be a better choice for making bio-preparations and minimize the risk of drawbacks and promote environmental protection. Endophytic bacteria can invade and colonize within plants in such a way that they are specific and remain intact. Moreover, strains of endophytic bacteria can enhance plant protection from phytopathogens and improve plant immunity.

The prominent abilities of endophytic bacteria to improve plant growth and minimize pathogen infestation via direct and indirect mechanisms [18,19] are attracting scientists globally. Direct mechanisms are set up to apply to those bacterial traits that directly promote plant growth. They include the production of Indole-acetic-acid (IAA), 1-aminocyclopane-1 carboxylic acid (ACC), and Gibberellic acids (GA), and also facilitate the uptake of fixed nitrogen, siderophore, phosphorus, and zinc production. Under indirect mechanisms, endophytic bacteria improve plant growth by acting as biocontrol agents. In addition, many endophytic bacteria have been reported to produce compounds classified as secondary metabolites for inhibition of pathogens, and they also adopt different mechanisms in order to produce cell wall degrading enzymes, antibiotics, competition and induced systemic resistance (ISR) [20].

Herein, we describe the comparative information of endophytic bacteria inhibit the rice plant, as well as their plant-growth-promoting traits and their biocontrol activity against pathogens; we also describe the innovative techniques and bioinformatics tools that may help to identify the key endophytes communities and plant growth-promoting genera. In addition, we discuss their phyla, classes, orders, families, genus, and species abundances in the different genotypes of rice plants.

## 2. Endophytic Bacterial Diversity in the Rice Plant

**Figure 1:**
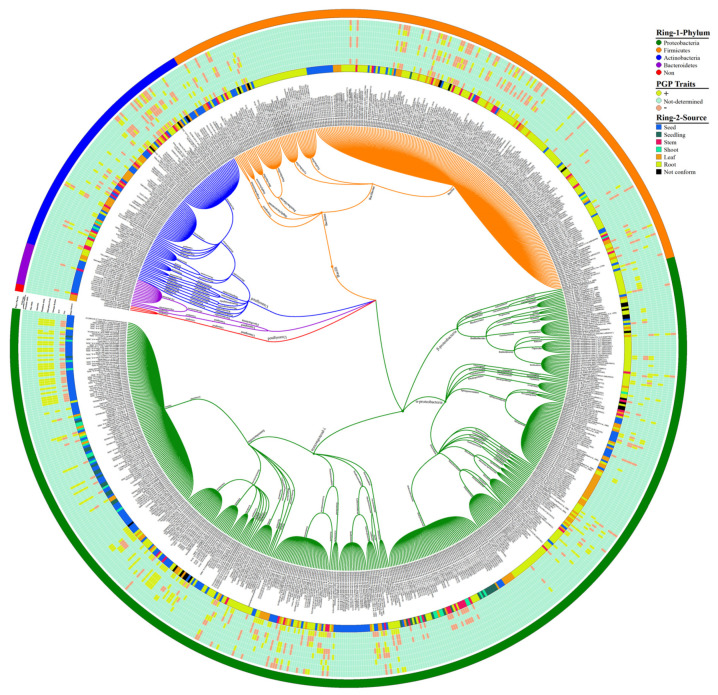
Rice endophytic bacterial communities. Each specified color in Ring-1 represents each phylum, and Ring-2 illustrates the source of bacterial communities from rice plants. An intermediated portion between Ring-1 and Ring-2 shows the plant-growth-promoting (PGP) attributes produced by rice endophytic bacterial strains; the (+) symbol indicates the presence of PGP traits, whereas the (−) sign indicates the absence of traits. The eight Arabic digits between in brackets indicate the accession number of the National Center for Biotechnology Information (NCBI) and references for taxonomic classification of bacterial communities (see the Supplementary Materials Data 2 for high-resolution visualization of figure).

**Figure 2:**
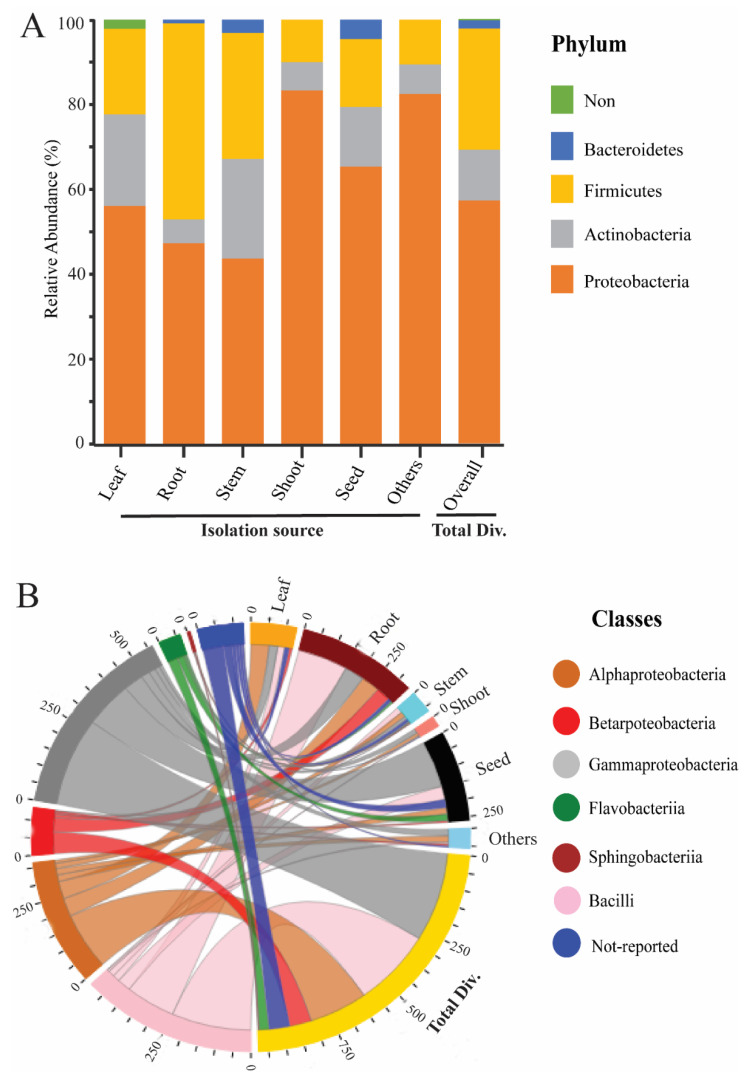
Relative abundance of rice endophytic bacteria diversity within rice tissues. (**A**) Endophytic bacteria diversity at the phylum level; the isolation sources were plotted on the horizontal axis, and relative percentage of phyla is visualized by the vertical axis, each taxon showed with specified color in stacked columns. (**B**) 3D chord diagram illustrates the rice endophytic bacteria diversity at the class level; each arc shows overlapping diversity within rice tissues; 3D visualization available on following link https://beta.rstudioconnect.com/connect/#/apps/42c60c75-8ff2-4dce-ad48-c1446c8ccb55/access, accessed on 5 September 2021 Total Div.: presenting the total diversity within rice tissues (leaf, root, stem, shoot, seed, and including other sources). Other: including isolation sources of rice (grain filling, tillage stages, and also not-conformed sources).

To study the endophytic bacteria taxonomic flora in the rice plant, we collected the prehistoric taxonomic data until 2020, with their plant-growth-promoting traits and their antagonism against phytopathogens, using the National Centre of biotechnology information (NCBI) PubMed (https://pubmed.ncbi.nlm.nih.gov/, accessed on 25 September 2020), Google Scholar (https://scholar.google.com/, accessed on 25 September 2020), and SciencDirect (https://www.sciencedirect.com/, accessed on 25 September 2020) web portals; after that, we plotted the taxonomic tree by writing a self-script in Python 2.7 [21] to convert the excel file into Newick format; the further Newick-format file was visualized and edited in iTOL version 5 [22]. Our data analysis showed 4 phyla, 6 classes, 22 orders, 46 families, and 74 genera of endophytic bacterial communities that have been mined from rice parts based on a culture-dependent method; complete details of taxonomic distribution are visualized in Figure 1. Moreover, our data analysis showed that the phylum Proteobacteria has a high abundance among rice tissues, while Bacteroidetes has a lower abundance (Figure 2A). To seek more details at the class level, we plotted the 3D-chord diagram to visualize the overlapping and diversity of classes within rice tissues. As a result, Gamma proteobacteria and Bacilli were more diversified and overlapped among rice tissues in contrast to Betaproteobacteria, Flavobacteria, and Sphingobacteria (Figure 2B).

The majority of the endophytic bacterial diversity was isolated from root portions that displayed more diverse communities. Many researchers isolated *Bacillus*, *Pantoea*, *Methylobacterium* and *Sphingomonas* genera, they are in high abundance with diverse species from the seed [23,24], root [25,26], and leaf part [27,28] of the rice plant (Supplementary Materials Data 1, Figure S1). Most species of *Rhizobium*, *Azospirillum*, *Burkholderia,* and *Herbaspirillum* were isolated from root tissues with distinct species [29,30,31,32], and *Xanthomonas*, *Flavobacterium*, and *Knoellia* genera were isolated from seeds (Supplementary Materials Data 1, Figure S1) [33,34]. These endophytes genera also have been identified after isolation from different plants, such as ben tree, (*Moringa peregrina*) [35], maize (*Zea mays*) [36,37], *Datura metel* (local name Devil’s Trumpet) [38], Kudouzi (*Sophora alopecuroides*) [39], Scots pine (*Pinus sylvestris*) [40], strawberry (*Fragaria ananassa*) [41,42], wheat (*Triticum aestivum* L.) [43], rose gum (*Eucalyptus grandis*) [44], sugarcane (*Saccharum officinarum*) [45], grapevine (*Vitis vinifera*) [46,47], poplar (*Populus deltoides*) [48], pepper (*Capsicum annuum*) [49], Huang-Qin (*Scutellaria baicalensis* Georgi) [50], cotton (*Gossypium hirsutum*) [51], potato (*Solanum tuberosum*) [52], cucumber (*Cucumis sativus*) [53], tobacco (*Nicotiana tabacum* L.) [54], peanut (*Echinopsis chamaecereus* “Lutea”) [55], tulasi (*Ocimum sanctum*) [56], and pea (*Pisum sativum* L.) [57]. Few studies have reported that bacterial genera are specific for seed colonization; for example, the *Pseudomonas putida* mutant during the secretion-system investigations displayed the reduction in their potential to colonize with maize seeds [58]. On the other hand, rice root endophytic bacteria are more diverse [25,59] compared to seed endophytes [60,61]; complete details can be visualized in Figure 1.

Seeds can also be colonized by bacteria existing on the surfaces of flower petals, fruits, and stems [62,63], and by additional pollen grains’ bacteria [64] that may be associated with ovules after pollination processes [65]. Root endophytic bacteria can be explored by using the soil surrounding plant roots or plant litter [36,66]. Moreover, endophytic bacterial diversity variation is associated with physicochemical properties of soil, as well as atmospheric conditions, cropping history, geographic location, and other agricultural practices [36,67,68]. In certain studies, it is suggested that root endophytic bacteria can mainly exist among the rhizosphere parts, and some of them can migrate through the xylem track to colonize the aerial parts [69,70].

Seed-borne pathogens can be persistent among new generations of plants. In the same way, seeds can also be harbored by endophytic bacteria that might be hereditary to new generations. Non-pathogenic bacteria have been categorized, from seeds and roots, parts of rice [25,29], maize [37], wheat [71], and other plants [37,72,73]. Many endophytes responsible for plant-growth-promoting traits (such as IAA, ACC, siderophore production, nitrogen fixation, phosphorus production, zinc solubilization, and ammonia production) have been isolated from leaves, seeds, stems, and roots of rice plants (Figure 1). These beneficial endophytes assist the host plants to maintain their growth and increase immunity against phytopathogens.

## 3. Factors Involving in Endophytic Bacterial Communities’ Variations

Endophytic bacterial communities strongly vary by environmental factors, numerous factors affecting endophytic bacterial diversity, such as host plant genotype, age of the plant, agricultural practices, and multi-kinds of nutrient availability, may help to increase bacterial diversity with diverse genera [74]. It is reported by Hardoim et al. [62] that the rice endophytes community varies with the growth stages of rice plants. The genotype of rice plants strongly influenced the endophytes community types [75]. It is well documented by Khaskheli el al. [25,59] that different rice cultivars planted in the soil containing same physiochemical properties can have distinct endophytic taxonomic composition and diversity. Thus, the soil physicochemical properties and different genotypes of plants can determine the endophytes communities’ composition. Numerous studies reported that the same genotypes of plant species were grown in different soil types having diverse endophyte communities with distinct taxonomic profiles [36]. Recently, climate conditions are influencing endophytic bacterial diversity composition and taxonomic variations [76,77]. For instance, Ren et al. [78] reported that rice endophytes’ bacterial community composition and their diversity are significantly affected by climate conditions. It is observed by Walitang et al. [79] that rice’s endophytic bacterial community diversity and diverse genera profile are influenced by salt concentration in the growth environment. Researchers reported that the resistant genotypes of different plant species against phytopathogens harbor a major portion of the endophytic bacterial community antagonistic to certain phytopathogens comparative to susceptible genotypes [80]. Andréa et al. [80] illustrated that the endophytes community was different from symptomatic and non-symptomatic samples of *Colletotrichum* spp. and *Paullinia cupana*, respectively. Hence, the selection of the endophytic bacteria community process is a very complex process, which is firmly controlled by the host plant tissues, it is well documented by Berendsen et al. [68] that plant roots play an essential role for the retrieval of microbiome community from soil.

## 4. Method for Isolation and Identification of Endophytic Bacteria

**Figure 3:**
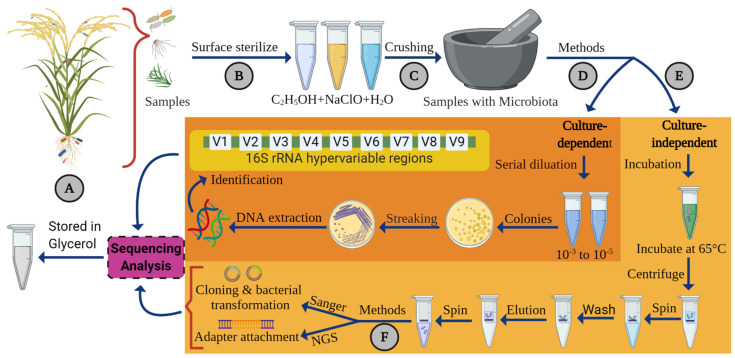
Methods of isolation and identification of rice endophytic bacterial communities: (**A**) samples collected from different parts of rice plants; (**B**) standard surface sterilization of the samples; (**C**) crushing of samples, followed by two methods for isolation, namely (**D**) culture-dependent method and (**E**) culture-independent method. (**F**) In culture-independent method, identification via Sanger sequencing or NGS; the figure was created with BioRender (https://biorender.com/, accessed on 25 August 2021).

A majority of plant endophytic bacterial communities have been isolated and identified through culture-dependent methods [23,24,25,26,59,60,75,81,82,83,84,85]. However, to study the endophytic bacteria diversity by using these methods, the bacterial strains must be culturable under in vitro conditions. For endophytes, bacterial strains isolated from plant samples are completely dependent on the isolation procedure. The most common track for the isolation of endophytic bacteria diversity from rice tissues using a culture-dependent method which can be visualized in Figure 3, but each study uses a different concentration of surface sterilization agent according to the sample size, shape, and thinness of the rice tissues [27,86]. The surface sterilization of rice samples was carried out by using sodium hypochlorite (NaClO) with different concentrations, and then the samples were washed with 70% ethanol solution at the end, to use sterilize distilled water for removing traces of chemical solutions from samples [60,87]. For the confirmation of the effectiveness of the surface sterilization procedure, the last washing water is plated on the culture medium. Moreover, culture medium is very important to explore the endophytic bacterial diversity; for rice endophytes bacteria, most of the studies used Tryptone soya agar (TAS) medium, which is also known as a Trypticase soy agar [31,60,86,88,89]. Endophytes bacteria populations are dependent on the type of growth media used to culture the bacteria from plant tissues, host plant growth environment, and surface sterilization method to plant tissues. Growth-medium selection highly affects the number and divers of endophytes bacteria communities; indeed, there is no a single growth medium which can fulfill the growth requirements and nutritional values of all bacteria [90]. However, the abundance of culturable microorganisms is less than 1% in the environment, and this may be due to their unknown nutrient requirements or in vitro conditions, or due to the fact that a few microbes’ cells cannot be culturable, even if their cells are visible under an in vitro environment [78,91].

Further culturable bacteria identification is based on their morphological, physiological growth habits, and biochemical and molecular techniques [92]. Several kinds of molecular markers were available for the identification of specific microbial taxa. Among them, the most famous 16S rRNA gene markers were used for bacterial phylogeny analysis and their taxonomic classification. For rice endophytic bacterial taxonomic classification, most of the researchers amplify the 16S rRNA gene by using 27F (forward primer) and 1492R (reserve primer) for the identification and the taxonomic position [24,25]. Currently, the taxonomic classification of prokaryotes is based on a combination of steps from phenotypic, chemo-taxonomic, and genotypic data; this process is commonly known as poly-phasic taxonomy [93]. These steps provide sufficient information for taxonomic classification for most phyla of bacteria, but these steps are not sufficient for the Actinobacteria phylum [94]. Accurate taxonomic classification of Actinobacteria phylum can be achieved by using whole-genomic sequencing (WGS) [95,96]. Further genomic data proceed through DNA–DNA hybridization (DDH) analysis to attained authentic taxonomic classification of Actino-bacterial species [97]. Moreover, average nucleotide identity (ANI), including multilocus sequence analysis (MLSA), is highly recommended for genetic taxonomic profiles [98].

The culture-independent methods inhibited the culture-dependent methods and provided fast and true endophytes bacteria taxonomic. The genomic DNA (gDNA) extraction from surface-sterilized plant tissues, after that selective culture-independent method, was used to identify the endophytes communities (whole procedure is visualized in Figure 3). Moreover, several fingerprinting techniques can rely on the gDNA to amplify the specific gene marker of microbes; the most common known marker is the 16S rRNA gene for bacterial diversity analysis [99]. Several fragments of the 16S rRNA gene were amplified through DNA fingerprinting techniques, such as temperature gradient gel electrophoresis (TGGE), Amplified rDNA Restriction Analysis (ARDRA), Terminal Restriction Fragment Length Polymorphism (T-RFLP), denaturing gradient gel electrophoresis (GGGE), and automated ribosomal intergenic spacer analysis (ARISA) [100,101,102,103]. The culture-independent method has been utilized to obtain sequenced data, using Sanger sequencing and next-generation sequencing (NGS). For the identification of rice’s endophytic bacterial diversity, many researchers used Sanger sequencing [26,104,105]. A few studies used NGS for rice leaves and root endophytic bacterial community analysis [106,107]. In recent year, NGS applications have been excessed more in microbiome data analysis to identify the endophytic bacterial diversity from potato, spinach (*Spinacia oleracea*), lettuce (*Lactuca sativa*), and from the roots and leaves of *Arabidopsis thaliana* [108,109]. This method has been used to sequence the whole genome of plant endophytes’ microbe diversity and assign their true taxonomic afflictions [110].

## 5. Mechanism of Plant-Growth Promotion by Endophytes

The endophytic bacteria have been revealed to indirectly or directly transmit beneficial effects to their host plant. They can directly support plants to enhance the availability of easily usable nutrients and by regulating growth substances (phytohormones), which can increase growth and development of plants under normal and abnormal conditions [111]. The indirect mechanism, the endophytic bacteria, improves plant growth by decreasing the level of ethylene in plants, producing antibiotics and compounds for the inhibition of pathogens, producing cell-wall-degrading enzymes, and stimulating systemic resistance [112]. However, the direct mechanism of endophytic bacteria facilitates the host plant by increasing the uptake of phosphorus and helps the plant in establishing the biological nitrogen fixation, zinc solubilization in roots, and phytohormones within plants (e.g., IAA, ACC, and GAs) [20]. Herein, we discuss and summarize these mechanisms below.

### 5.1. Direct Plant-Growth Promotion by Endophytes

An adequate amount of nutrients that are required for plant growth, which are typically deficient in soils, can be attained through endophytic bacteria. They have the potential to increase and provide certain kinds of nutrients to the host plant, such as nitrogen, iron, and phosphorus. Details of these nutrients and responsible processes and/or mechanisms are discussed below.

#### Biological Nitrogen Fixation

**Figure 4:**
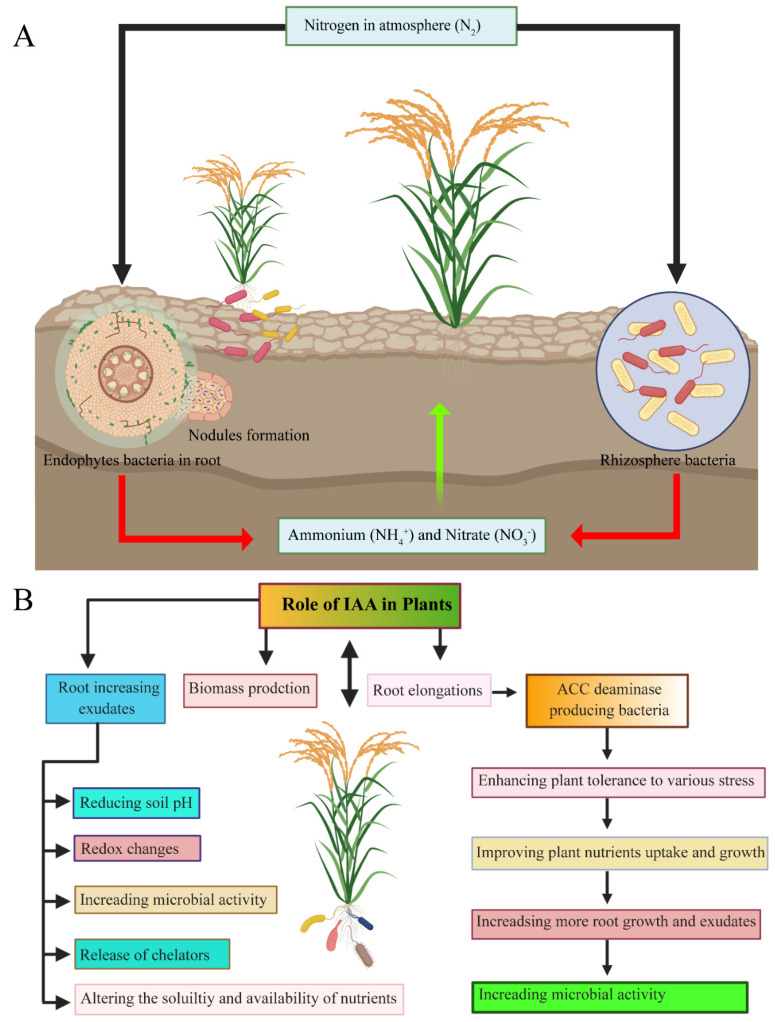
Direct plant-growth promotion by endophytic bacteria. (**A**) Process of biological nitrogen fixation from the atmosphere by beneficial bacteria. (**B**) Importance of Indole-acetic-acid (IAA) for plant growth. Figure was created by BioRender.

Endophytic bacteria, express nitrogenase activity, which can play a role to fix atmospheric nitrogen in an available form for their host plants. Nitrogenase is a highly conserved protein, and this enzyme is commonly found in all nitrogen-fixing bacteria, with sufficient proof proposing that lateral gene essential components for biological nitrogen fixation, according to Ivleva et al. [113]. Ambient dinitrogen exists more than 78% in the atmosphere that is unavailable for direct plant consumption without conversion to available ammonia form for plant uptake. Several rice endophytic bacterial species help to increase the nitrogen fixation, such as *Azoarcus* sp. BH72, *Herbaspirillum seropedicae*, *Burkholderia* sp., and *Gluconacetobacter diazotrophicus*, have been reported to increase N_2_ fixation among the host plant and improve plant biomass under aseptic conditions [60,114]. There are three main processes for atmospheric nitrogen conversion into a usable form for plants: (i) the formation of the nitrogen oxides in the atmosphere from atmospheric nitrogen; (ii) the industrial formation of ammonia via processing the atmospheric nitrogen under catalytic processes in the presence of a high temperature (300–500 °C); and (iii) biological nitrogen fixation via microorganisms by utilizing complex procedures with the help of enzymes, such as nitrogenase, that effectively convert atmospheric nitrogen into ammonia [115,116] (see Figure 4A).

Biological nitrogen fixation is the key process that is fixing about 60% atmospheric nitrogen on the earth. Moreover, it is more environmentally friendly and economically valuable than industrial chemical fertilizers [116,117]. Nitrogen fixation takes place among non-leguminous crops by Plant-Growth-Promoting Rhizobacteria (PGPR) formally, known as diazotrophs [118]. The nitrogen fixation mechanism comprises the activity of nitrogenase, which is encoded by *nif* genes [119]. In bacteria, the nitrogen-fixation system has variations among different bacteria depending on the environments and, importantly, their growth rate [120]. Endophytic bacteria colonize within plant tissues/parts (e.g., leaves, stem, roots, and even reproductive organs), have the ability to fix atmospheric nitrogen, promote the plant growth, and protect plants from pathogens infestation [121,122,123]. Previous studies showed that the endophytic bacteria from rice, wheat, and maize, such as species of *Burkholderia* genus, *Enterobacter asburiace*, and *Herbaspirillum seropedicae*, and including other bacteria species of the *Azotobacter* genus, which are potential biological nitrogen fixers [1,60,124]. However, Carrell and Frank [125] have noted that the *G. diazotrophicus* strain has a great potential for nitrogen fixation, and it forms a symbiotic relationship with sugarcane and pine plants.

### 5.2. Availability of Phosphate

Phosphorous is also an essential micronutrient which is vital for enzymatic reactions after nitrogen that are necessary for many physiological processes in plants [126]. However, much of the soil’s phosphorous is not available in suitable forms that can be absorbed by plants. Plants can only absorb the soluble forms, mono- and dibasic phosphate. Besides, it is estimated that 75% of phosphorus application during agricultural practices in the form of fertilizer becomes unavailable for the plants from soil [127]. Endophytic bacteria can improve phosphorus supply for plants by solubilizing precipitated phosphates, using mechanisms such as acidification, chelation, exchange of ions and organic acid production. The rhizosphere species also belongs to such microorganisms that provide the iron soluble form to plants by processing siderophore products and have been isolated from rice, cucumber, cotton, peanut, sorghum (*Sorghum bicolor*), and maize [128,129,130]. Several bacterial communities were isolated from plant compartments and rhizosphere, have a great ability to provide a soluble form of the phosphorus to plant roots by mineralization of the organic and/or inorganic phosphate solubilization via acid production [131,132].

In addition, endophytes play an important role in the adsorption and also fixation of phosphate through assimilating solubilize phosphorus under phosphate-limiting conditions [133]. Hence, these bacteria may serve as a drain to supply the host plants with phosphorus when they require it. Characteristic of phosphate solubilization processes is usually encountered in endophytic bacteria. For example, about 59–100% of endophytes diversity were isolated from strawberry, rice, soybean (*Glycine max*), maize, cactus, and other legumes crops showed the potential of mineral phosphate solubilization [134,135]. Many rice endophytic bacterial strains effectively have been studied for phosphate solubilization (Figure 1), e.g., *Burkholderia* sp. strain BRRh-4 inoculation in rice seedling significantly enhanced the growth and grain yield of rice variety [136,137,138].

### 5.3. Phytohormones

Endophytic bacteria can boost the accumulation of nutrients and enhance the metabolism of host plants by generating phytohormones. Recent research investigating the colonization of phytohormones producing endophytic bacteria with the host plant increased plant nutrient absorption and biomass of plants [74]. There are five famously known types of phytohormones, abscisic acid (ABA), auxin, gibberellins (GAs), cytokinins (CKs), ethylene (ET) and Indole-3-acetic acid (IAA), these two hormones (IAA and ET) are the most important participants in building plant–bacterial interactions [139]. During the last decade, several rice endophytic bacteria showed that they have the great potential for phytohormone production [29,140].

#### 5.3.1. Auxins

Several microorganisms produce auxin as the secondary metabolite, and many of them are isolated from the inner and outer parts of plants [141]. Many studies showed that the microorganisms produce IAA under peptone or tryptophan precursor availability. The IAA plays a multi-functional role in plant-growth promotion, such as cell division and enlargement, differentiation of tissues, primary root elongation, root inhibition, and physiological functions [142]. The most common genera of such bacteria are *Alicaligenes faecalis*, *Azospirillim*, *Pseudomonas*, *Xanthomonas*, *Enterbacter cloacae*, *Bradyrhizobium japonicum*, and *Bacillales*; they have displayed auxin production potential and enhance plant growth [143,144]. Various rice endophytic bacteria have been isolated from stem, roots, and leave tissues of different rice genotypes and have the potential to produce IAA in different amount of concentrations (see Figure 1) [29,59,140,145]. IAA biosynthesis is not the only way in which endophytic bacteria can enhance the growth of host plants. Certainly, its reverse process, the IAA catabolism/degradation, may also play an important role in boosting plant growth and its production. Leveau et al. [146] reported that *Pseudomonas putida* strain 1290 displayed an effective role in IAA degradation to enhance plant growth, such as elongation of the radish (*Raphanus sativus*) roots through eliminating the inhibitory effects of exogenous IAA. In the presence of tryptophan, which is a precursor of IAA, the bacteria also produce IAA. Nevertheless, it also has been described that the development of IAA by *P. putida* strain 1290 did not show the same deleterious impact on the radish roots compared to other high amounts shown by IAA-producing strains. The author proposed that its dual status mechanism, which includes the potential of both IAA production and its degradation, which enables this bacterial strain to produce the IAA total amounts, which can provide their beneficial effect to the host plant. Currently, several endophytic strains have been isolated and tested for the production of IAA and also showed a significant effect on plant-growth attributes, such as root and shoot elongation, and root and shoot fresh and dry weight; they also enhance chlorophyll contents in rice, soybean, and barley [27,147,148], as shown in Figure 4B.

#### 5.3.2. Cytokinins

Many studies reported that certain beneficial endophytic bacteria can produce cytokinins (CKs) during interaction with host plants; CKs are N6-substituted amino purines that promote plant growth by activating cell division in roots and shoots parts of the plant [149]. The CKs are well-recognized phytohormones that promote plant roots’ growth, development, and release by non-pathogenic microorganisms adhering to plant roots [150]. The adenine precursor synthesis for CKs’ production by a common synthetic pathway in microorganisms such as *Azotobacter* sp., *Corynebacterium fascians*, and *Rhizopogon roseolus* highlighted the potential of the microbes for use in CKs’ synthesis [151,152]. A total of 90% of bacterial strains can produce cytokinin-like substances; these strains are isolated from the rhizosphere of different crops [116]. In vitro production of cytokinin-like compounds on growth media by different isolated bacterial strains from pine and *Sambung nyawa*, seedling roots strengthen the application of microbes in crops for improving plant growth and immunity [153]. The strains *Bacillus* and *Pseudomonas* that are isolated from various varieties of plants, such as rice, barley, canola (*Brassica napus* L.), soybean, and *Arabidopsis*, can produce these compounds. Endophytic *Bacillus* spp. have a great ability to produce CKs, which act as growth regulators [154].

#### 5.3.3. Gibberellins

Gibberellins’ (GAs) composition consists of a type of terpenoids commonly referred to as iso-prenoids, which contain 20-carbon atoms, but in the active form, it contains 19-carbon atoms and also plant hormones. Additionally, 130 molecules are reported in these compounds [155]. GAs play an important role in stem elongation, seed germination and growth, and parthenocarpy, and more prominent action was observed during internode elongation. Many rice endophytic bacterial communities, such as *Azospirillum brasilense*, *Acetobacter*, *Arthrobacter*, *Agrobacterium*, *A. lipoferum*, *Bacillus* sp., *Pseudomonas*, *Clostridium*, *Rhizobium*, *Flavobacterium,* and *Xanthomonas*, have great potential to synthesize gas [156]. *Acinetobacter calcoaceticus* can produce GAs and its application in Chinese cabbage and crown daisy promoted the growth rate, as compared to non-inoculated crops [157]. It is reported by Ishak et al. [158] that *B. subtilis* strain LKM-BK is an endophytic bacterial strain isolated from surface-sterilized tissue of *Theobroma cacao* plants, which can produce GAs in certain concentration and further compounds of trans-zeatin. These findings showed the use of endophytic bacterial strains for plant-growth promotion and pathogen suppression is an applicable and result-oriented approach in agriculture development globally.

#### 5.3.4. Ethylene and 1-Aminocyclopropane-1-Carboxylate Deaminase

Ethylene (ET) is a phytohormone and is broadly well-known for enhancing the process of ripening in different fruits and flower senescence. The ET plays an important role in plant developmental stages, such as ripening and abscission of fruit, flowering, petal, leaf senescence and abscission, root growth, and hair establishment [159]. It has been demonstrated by Dodd et al. [155] that ET also plays a role in root hair promotion, but it inhibits lateral root formation and primary root prolongation. Alleviated ET production was observed in plants against stresses, such as drought, pathogen attack, waterlogging, soil salinity, heat and cold stress, and high concentration of heavy metals [155,160]. ET is well known as a stress indicator hormone due to its synthesis under abiotic and biotic stresses under pathogen attack and drought conditions. ET in the planta produces 5′-deoxy-5′methylthioadenosine (MTA) and ACC by converting the S-adenosylmethionine (SAM), using enzymatic processes [161].

Several rhizospheres and endophytic bacteria contain enzymes of ACC deaminase, e.g., *Pseudomonas*, *Bacillus*, and *Achromobacter*, and also in very famous fungi, such as *Trichoderma* sp. Plant roots release ACC enzymes; these microbes transfer this enzyme to other parts of plant tissue and convert it to α-ketobutyrate and ammonia, as reported by Glick et al. [162]. Moreover, various studies have reported the ACC activity in rhizosphere, endophytic, and diastrophic bacteria that belong to a wide range of genera, ad well as their potential for growth promotion in the presence of several stress conditions, such as in maize [163], tomato (*Solanum lycopersicum*) [164,165], and wheat [166]. Ethylene protects the plant from pathogen attaching via altering the virulence signaling molecules to defend against the pathogen. These results indicating that a reduction in ACC and ET level in planta indicate a decreasing level of stress. Inoculation of bacteria with the ability to produce enzyme ACC deaminase in plants, which can protect plants from stresses induced by the high concentration of heavy metals, drought conditions, over flooding, waterlogging, pathogen attacks and organic toxic compounds [160,162,167].

#### 5.3.5. Abscisic Acid

Abscisic acid belongs to phytohormones and comprises 15-carbon compounds that have similarities with ethylene. It plays an important role in seed development and particularly in maturation stages; activates plant defense mechanisms against stressors, such as salt stress, drought, a toxic metal, etc.; and plays a vital role in stomatal conductance. All of these features enhance its importance for plant-growth promotion [168]. In vitro ABA production by different genera of endophytes and free-living bacteria, such as *Bradyrhizobium japonicum* and *Azospirillum* sp., promotes its synthesis [169]. For instance, *A. thaliana* was inoculated with *Azospirillum brasilense* strain (Sp25), and the alleviated ABA level was observed under inoculated and non-inoculated plants. Moreover, ABA was reported to inhibit CK synthesis and enhance plant growth via modulating the CKs consortium [170]. It also experimentally proved that ABA increases shoot and root elongation via providing relief to the plants against stressors [169].

## 6. Indirect Plant-Growth Promotion by Endophytes

### 6.1. Biocontrol of Plant Pathogens by Endophytic Bacteria

**Figure 5:**
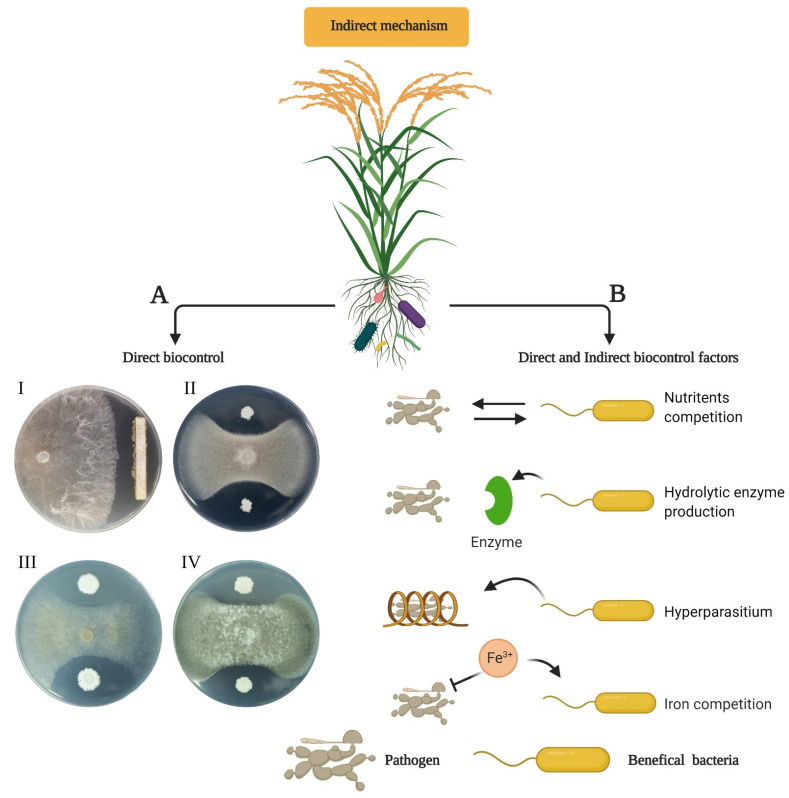
Indirect plant-growth promotion via biocontrol of phytopathogens. (**A**) Side showing directly biocontrol potential against fungal pathogens. (I) *Rhizoctonia solani*, the pathogen of rice sheath blight (RSB); (II) *Fusarium oxysporum*, the causal agent of rice sheath rot and seedling rot; (III) *Alternaria alternate*, causal agent of leaf spots and blight in several plant parts; and (IV) *Botrytis cinerea*, causal agent of several horticulture crops which are responsible for casing botrytis bunch rot (BBR). (**B**) Side showing indirect or direct factor role of beneficial bacteria for growth promotion by competing against phytopathogens in several ways, as shown in the figure. The figure was created by using BioRender.

The endophytic bacterial strains have great biocontrol potential against pathogens, including soil-borne pathogens and phytopathogens [171]. Still, many laps exist in understanding the biocontrol mechanism of phytopathogens and the application of the endophytic bacterial strains to suppress pathogen infestation and support the plants’ ability to improve their immunity. The biocontrol mechanism of plant pathogens comprises a multifactor interplay to suppress/inhibit pathogens, such as nutrient competition, antibiotic production, and ISR against pathogens. The endophytic bacteria mediate several mechanisms for biocontrol, and ISR is the potential mechanism that was reported in planta. The endophytic bacterial community can be observed through microscopic examination and also morphological changes, also examined within the plants to investigate the ISR. For instance, several *Bacillus* spp. have been reported by Melnick et al. [172], to colonize cacao plants and showed potential to suppress symptoms of the black-pod-rot disease and displayed effective biocontrol against *Phytophthora capsici* pathogen. Several studies reported that rice endophytic bacteria have highly potential against various phytopathogens (Table 1 and Figure 5). During in vitro assay, it was revealed that 18 strains out of 37 endophytic bacterial strains from *Techona grandis* and *Samanea saman* Merr L. have produced inhibiting compounds against *Candida albicans* [37,173,174,175] and many *Bacillus* and *Streptomyces* endophytic bacterial strains were also isolated from rice, wheat, maize, rice, and garlic that displayed the strong potential of antifungal activity against pathogens *F. oxysporum*, *F. graminearum*, *R. solani*, *M. kuwatsukai*, *B. cinerea*, *R. cerealis*, and *G. graminis* [175].

### 6.2. Antibiosis

Antibiotics producing bacteria are largely identified and characterized for antibiotic biosynthesis and regulation under different environments and they are designated as the best biological control agents (BCAs). Antibiotic biosynthesis is regulated by many environmental factors, such as high or low temperature, pH variations, the concentration of different metal ions, etc. [180]. Even so, different bacterial strains require different environmental factors [181]. Therefore, the signaling mechanism and interaction between pathogens, plants and bacteria for antifungal traits’ modulation acquire tripartite interaction systems [182]. Previous literature revealed that a strong colonization interaction between bacterial strains and plant tissues is the key to antibiotic production [183]. The primary and secondary metabolites production is related to the bacterial-strain growth rate, it is reported by Haas and Défago [184], that bacterial growth rate boosts the secondary metabolites production. Bacteria also have unique features, such as biofilms, that play an important role in quorum sensing. The biofilm-containing bacteria play a vital role in plant protection against pathogen attack by forming a skin-like cover around plant roots and releasing antimicrobial compounds that abandon pathogen infestation [185].

### 6.3. Signal Interference

Primarily, many pathogens attack the plant cell wall to carry out their action. During this interaction, different kinds of exo-enzymes were detected by quorum sensing. Pathogens act very smartly to inactivate these exo-enzymes with N-Acyl homoserine lactones (AHL) molecules, and this whole process is known as signal interference [186]. There are only two kinds of classes of AHLs, which have been identified as successful activators, designated as AHL-acylase, which play an important role in breaking amide linkage and promoting abiotic factors [187]. It was revealed that AHL generates signals to release volatile organic compounds (VOCs) and suppress disease attacks in oilseed rape crops during pathosystem investigation of *Verticillium dahlia* and production of VOCs and antifungal metabolite (AFMs) demonstrated by Müller et al. [188] in *Serratia plymuthica* strain HRO-C48. Dandurishvili et al. [189] demonstrated that *S. plymuthica* strain IC1270 and *P. fluorescens* strain B-4117 generate VOC compounds which might help to control pathogenic *Agrobacterium* and suppress the tomato crown gall disease infestation. Moreover, Chernin et al. [190] reported that these strains produce VOC compounds and inhibit the transcription of AHL synthase genes.

### 6.4. Predation and Parasitism

The endophytic bacterial strains might adopt a predation and/or parasitism mechanism to control fungal pathogen infestation. The microorganisms or endophytes can synthesize or release cell-wall degrading enzymes such as chitinase, glucanase, and cellulose [191,192]. The endophytic bacteria kill fungal pathogens via disrupting fungal cell-wall with the excretion of cell-wall degrading enzymes, this mechanism is known as predation and parasitism. A similar mechanism was also found in *Serratia marscens* to control the pathogen attack described by Ordentlich et al. [193]. The *Curtobacterium flaccumfaciens* strain was isolated from the citrus plant and showed predation and parasitism mechanisms to prevent *Xylella fastidiosa* pathogen infestation [194].

### 6.5. Induced Systemic Resistance

The discovery of many PGPR free-living or endophytes in soil and those associated with compartments of plants such as leaves, roots, and plant reproductive parts, triggers induced systemic resistance (ISR) in the immune system of plants [195]. The chemical and pathogen application induces systematic-acquired resistance (SAR) during the plant defense system and SAR induction is responsible for fluctuations in salicylic acid (SA) and pathogenesis-resistance protein (PP) production [196]. Plant basal resistance is enhanced via ISR under different environmental conditions against many pathogens of the plant [197]. For instance, the *P. fluorescens* strains WCS365 and WCS417R are well-known as the inducers of broad-spectrum resistance in plants [198,199]. Previous studies have reported that inoculated plants are more efficient in defense and respond rapidly to insects as well as pathogen attacks, leading to improved protection ability of plants. They have also described how ISR microbes activate systematically induced resistance in plants and their distant parts, such as leaves.

Furthermore, it has been well documented by Van Loon and Bakker [200] that *Pseudomonas* strains release a few types of compounds, such as psuedomanine, SA, and siderophore, which ultimately increase the defense system in radish plants. Inoculated *Arabidopsis* with Actinobacteria endophytes strains upregulated the defense pathways and protected the plants against pathogens *F. oxysporium* and *E. caratovora*. The resistance pathway against *F. oxysporium* is dependent on SAR and against *E. caratovora* resistance driven through ET or SA [196]. In order to colonize with their host plant, bacteria must defeat their host defiance response [101].

## 7. Implementation of Bioinformatics in Endophytes Diversity Analysis

Applications of new bioinformatics tools help to analyze the microbiome diversity and interpret the results perfectly and meaningfully. Quantitative Insights Into Microbial Ecology (QIIME 2) is a bioinformatics pipeline that uses decentralized microbiome diversity analysis with different packages’ plug-ins [201]. QIIME 2 is a completely open-source package that starts the analysis of raw DNA sequence data and finishes with high-quality figures and statistics for publications. Each plug-in package has its function in QIIME 2, such as UCLUST, Python Nearest Alignment Space Termination (PyNAST), q2-feature-classifier, and PyNAST, which are used for aligning the sequences [202]; then UCLUST can cluster the millions of sequences and clusters them into Operational taxonomic units (OTUs) based on sequence similarity. Taxonomic can be assigned by using q2-feature-classifier by running the Greengene or Ribosomal Database Project (RDP) Classifier [203]. These tools are practically well applied to several rhizosphere microbes of plants and the earth microbiome [204,205], but no one has applied them to culture-dependent rice endophytic bacteria diversity. Most researchers cluster sequence at 97% into OTUs [204,206,207], which was proposed by Edgar Robert [206], but a new study proposed [208] to enhance the accuracy of taxonomic affliction at the species level by increasing the optical identity level of sequence clustering at 99–100% into OTUs for the V4 hypervariable region of 16S rRNA gene. Applications of these tools are a very simple and rapid way to assign taxonomic. For the application of beneficial bacteria for plant-growth-promoting assays, firstly we need to characterize their plant-growth-promoting traits under in vitro conditions in order to provide a clue about their growth-promoting potential. To characterize these traits is a laborious and time-consuming task to analyze the beneficial bacteria for applications in the form of synthetic applications. One possible track to predict these traits by using a bioinformatics tool was experimentally proved by Zhang et al. [205]. One study predicted the nitrogen-fixation bacteria via a FAPROTAX bioinformatics package [209] and further designed their synthetic communities based on nitrogen-fixation bacteria and inoculated them into rice roots for growth promotion. Applications of these communities increase the rice growth parameters compared to un-inoculated plants. Application of these tools is possible and reliable for finding beneficial bacteria.

## 8. Endophytes-Induced Bioactive Compounds

The endophytic bacteria play a very essential role in bioactive compounds’ (BCs) production. Various kinds of BCs have been produced by endophytic bacteria, such as alkaloids, flavonoids, quinols, polyketones, phenols, peptides, and terpenoids [210,211], and they have been applied in industrial, medical, and agricultural fields for the production of drugs to treat numerous diseases [212,213]. Several endophytes also release specialized biologically and metabolite active compounds [214]. Moreover, endophytic bacteria associated with ethnomedicinal plants act as a possible origin of bio-products, and their applications under oxidative stress play a role in producing a new bioactive agent [215]. More than 80% of the natural drugs as a source of BCs and secondary metabolites are produced by the endophytes of medicinal plants [216]. Endophytic microorganisms are the depot of precious secondary metabolites that may act as a unique and excellent provenance of drugs for antimicrobial, antidiabetic, anti-arthritic, anti-insect, anticancer, and immunosuppressant action [210]. Up until now, just a few plants have been studied for their endophytes microbial diversity and capacity to produce bioactive compounds and secondary metabolites. The exposing of these compounds’ and metabolites’ properties by various endophytes is a significant alternative source to defeat by enhancing the level of drug resistors in several pathogenic communities [210].

## 9. Conclusions and Prospects

The current article retires the compressive data of endophyte communities associated with rice plants. Metadata analysis insightfully proposed that 4 phyla, 6 classes, 22 orders, 46 families, and 74 genera of endophytic bacterial communities have been identified from rice parts based on culture-dependent and culture-independent methods. This tiny portion of identified endophytes diversity creates a huge gap between known endophytes communities. Traditional culture-independent methods need to be combined with advanced techniques, such as NGS, which is a rapid technique for the identification of microbes. The application of this method needs to increase at a broad level and its results are comparatively better than old methods.

Moreover, the culture-dependent method needs to be revised with their advanced materials, such as media composition, or the use of different types of media at the same time for microbe’s diversity to explore their diverse microbes’ communities. The 16S rRNA marker gene is sufficient to allot the taxonomic profile for most bacterial phyla, but it is not reliable for the Actinobacteria phylum taxonomic profile due to its division into subclasses and suborders. The whole genomic sequence is the best way to allot the taxonomic profile of the Actinobacteria phylum. Moreover, the next-generation sequence raises more interest in taxonomic classification, and this method increases the accuracy of the taxonomic classification of microbes. Implementation of bioinformatics tools in endophytic bacterial diversity may enhance the results speed and quality, allot the true taxonomic profiles, and reduce time consumption.

The application of endophytes with plant-growth-promoting traits and with inhibitory potential against phytopathogens have been a primary purpose of sustainable agriculture. The application of endophytic bacteria in the form of bio-fertilizers and bio-pesticides can be improved if we elaborate on the mechanism of endophytic bacteria interact with their host plant. How endophytes colonize with their host plant, and which kind of substrate is provided by the host plant and how endophytic bacteria utilize them as a source of nutrients, etc., need to explored for a better understanding and for their bio-fertilizer application at the commercial level for organic agricultural production. This can be achieved when we have clear and vast details about the bacterial genes uttered in the plant rhizosphere. A few studies have been performed in this field, but their collected information is not enough.

Hence, a compressive analysis of bacterial transcriptomes expressed in the plant will focus on their endophytic bacterial communities’ lifestyle, which is hidden in the plant endo-sphere parts. Attaining these arguments in the form of a challenging task, we notes that it is incredibly hard to find eminent attributes of RNA transcripts from growing bacteria in their host plant. This job is further elaborated by the low cell density of these endophytic bacteria sustained in their host-plant parts, which react to activating plant defense responses. Therefore, it is very hard to obtain adequate high-quality bacterial RNA for transcriptome analysis. However, using advanced techniques, such as RNA-seq, which can efficiently recognize both rich and rare transcripts, these kinds of analyses are now possible but need to be implemented for these analyses at broad levels. In addition, meta-transcriptome analysis of bacteria, using advanced techniques, provides a clue about their gene abundances and their profiles and would be stronger to expose their endophytic communities’ activity, which attracts their host plants for their development and nutritional benefits.

Lastly, as the rice endophytic bacterial diversity and their taxonomic afflictions have been poorly analyzed, there is the prospect of discovering novel endophytic bacterial strains with beneficial traits from unexplored genotypes of rice islands. Climatic conditions highly affect rice plants, and changing weather conditions develop abiotic and biotic stress that has an extreme effect on endophytes diversity. The identification of these novel bacteria with unique features would require an amalgamation of culture-dependent and culture-independent methodologies.

## Figures and Tables

**Table 1:** Rice’s endophytic bacteria’s antagonism potential against various kinds of phytopathogens.

Endophytes	Host	Antagonistic Activity	References
*Bacillus wiedmannii* strain C-1CL-2	Japonica Rice (*Oryza sativa* L.) cultivar Xiushui 48	*Fusarium graminearum* and *Rhizoctonia solani*	[25]
*Bacillus wiedmannii* strain C-1CL-4		*Fusarium graminearum* and *Rhizoctonia solani*	
*Bacillus altitudinis* strain C-1W-5		*Magnaporthe oryzae*	
*Lysinibacillus fusiformis* strain C-1WF-3		*Fusarium moniliforme*	
*Fictibacillus phosphorivorans* strain C-1Y-9		*Fusarium graminearum* and *Rhizoctonia solani*	
*Fictibacillus phosphorivorans* strain C-1Y-10		*Magnaporthe oryzae*	
*Fictibacillus phosphorivorans* strain C-1Y-13		*Fusarium moniliforme*	
*Fictibacillus phosphorivorans* strain C-1Y-16		*Fusarium moniliforme*	
*Fictibacillus phosphorivorans* strain C-1Y-17		*Fusarium graminearum*, *Fusarium moniliforme* and *Rhizoctonia solani*	
*Bacillus altitudinis* strain C-2B-2 1	Rice (*Oryza sativa* L.) cultivar Y-003	*Rhizoctonia solani*	
*Bacillus altitudinis* strain C-2D-1 1		*Rhizoctonia solani*	
*Bacillus aryabhattai* strain C-2HW-1		*Fusarium graminearum* and *Rhizoctonia solani*	
*Lysinibacillus mangiferihumi* strain C-2HW-3		*Fusarium moniliforme*	
*Bacillus marisflavi* strain C-2LY-2		*Fusarium graminearum* and *Rhizoctonia solani*	
*Bacillus marisflavi* strain C-2LY-5		*Fusarium graminearum* and *Rhizoctonia solani*	
*Bacillus aryabhattai* strain C-2LY-6		*Fusarium graminearum*, *Fusarium moniliforme* and *Rhizoctonia solani*	
*Bacillus altitudinis* strain C-2RO-1		*Fusarium graminearum*, *Rhizoctonia solani* and *Magnaporthe oryzae*	
*Bacillus altitudinis* strain C-2RO-3		*Fusarium graminearum*, *Rhizoctonia solani* and *Magnaporthe oryzae*	
*Bacillus altitudinis* strain C-2RO-4		*Rhizoctonia solani*	
*Bacillus altitudinis* strain C-2S-1		*Rhizoctonia solani*	
*Bacillus altitudinis* strain C-2SN-3		*Fusarium moniliforme*	
*Bacillus cereus* strain C-2W-1		*Fusarium moniliforme*	
*Bacillus tequilensis* strain C-2W-3		*Rhizoctonia solani*	
*Bacillus indicus* strain C-2Y-1		*Fusarium graminearum* and *Rhizoctonia solani*	
*Bacillus marisflavi* strain C-2Y-2		*Fusarium graminearum*, *Fusarium moniliforme* and *Rhizoctonia solani*	
*Bacillus marisflavi* strain C-2Y-6		*Fusarium graminearum* and *Rhizoctonia solani*	
*Bacillus cereus* strain C-3CL-6	Rice (*Oryza sativa* L.) cultivar CO-39	*Fusarium moniliforme*	
Bacillus toyonensis strain C-3CL-7		*Rhizoctonia solani*	
*Bacillus wiedmannii* strain C-3CL-8		*Fusarium graminearum* and *Rhizoctonia solani*	
*Lysinibacillus mangiferihumi* strain C-3F-5		*Fusarium moniliforme*	
*Bacillus altitudinis* strain C-3R-1		*Fusarium graminearum* and *Rhizoctonia solani*	
*Bacillus altitudinis* strain C-3R-3		*Fusarium graminearum*, *Rhizoctonia solani* and *Magnaporthe oryzae*	
*Bacillus altitudinis* strain C-3R-8		*Fusarium graminearum* and *Rhizoctonia solani*	
*Bacillus cereus* strain C-3R-9		*Fusarium moniliforme*	
*Bacillus altitudinis* strain C-3R-10		*Fusarium graminearum*, *Rhizoctonia solani* and *Magnaporthe oryzae*	
*Cupriavidus metallidurans* strain C-3SP-3		*Magnaporthe oryzae*	
*Paenibacillus cucumis* strain C-3T-7		*Fusarium moniliforme*	
*Bacillus altitudinis* strain C-3WA-8		*Rhizoctonia solani* and *Magnaporthe oryzae*	
*Bacillus marisflavi* strain C-3Y-2		*Fusarium graminearum* and *Fusarium moniliforme*	
*Bacillus marisflavi* strain C-3Y-5		*Fusarium graminearum*, *Fusarium moniliforme* and *Rhizoctonia solani*	
*Fictibacillus phosphorivorans* strain C-3Y-10		*Magnaporthe oryzae*	
*Bacillus* sp. (in: Bacteria) strain CPS003	Rice *Oryza sativa* L. var. indica cv. RD41	*Bipolaris* sp. isolate KPS3, *Bipolaris* sp. isolate KPS5, *Curvularia* sp. isolate KPS41, *Nigrospora* sp. isolate KPS45, *Fusarium* sp. isolate KPS91 and *Curvularia* sp. isolate KPS102	[176]
*Bacillus* sp. (in: Bacteria) strain CZR007			
*Bacillus* sp. (in: Bacteria) strain CZS004			
*Bacillus* sp. (in: Bacteria) strain CZS006			
*Streptomyces endus* strain OsiSh -2	Rice (*Oryza sativa* L.) cultivar Gumei 4	*Magnaporthe oryzae*, *Colletitrichum karstii*, *Colletitrichum siamense*, *Colletitrichum camelliae*, *Colletitrichum frocticola*, *Colletitrichum glosporioides*, *Valsa mali*, *Guignardia bidwellii*, *Physalospora piricola*, *Botryosphaeria ribis*, *Rhizopus nigricans*, *Phomopsis vexans*, *Cladosporium fulvum*, *Stempblium consortiale*, *Botrytis cinerea*, *Colletrichum orbiculare*, *Helminthosporium maydis*, *Nigrospora oryzae* and *Rhizoctonia solani*	[140,145]
*Bacillus velezensis* strain YC7010	Rice (*Oryza sativa* L.)	*Fusarium fujikuroi*	[177]
*Bacillus velezensis* strain YC7007		*Fusarium fujikuroi* KACC 44022, *Magnaporthe grisea* isolate KACC 40415, *Bipolaris oryzae* isolate KACC 40853, *Rhizoctonia solani* isolate KCTC 40101, *Sclerotinia sclerotiorum* isolate GSCC 50501, *Botrytis cinerea* isolate KCTC 6973, *Fusarium oxysporum* isolate KCTC 16909, *Botryosphaeria dothidea* isolate GSCC 50201, *Pythium ultimum* isolate GSCC 50651 and *Alternaria panax* isolate KACC 42461	
*Enterobacter asburiae* strain VWB1	Rice (*Oryza sativa* L.) cultivar rex	*Fusarium oxysporum*	[178]
*Pantoea dispersa* strain VWB2		*Fusarium oxysporum*	
*Pseudomonas putida* strain VWB3		*Fusarium oxysporum*	
*Pantoea agglomerans* strain CT1	Rice (*Oryza sativa* L.) variety CT6919	*Pythium ultimum*	[179]
*Pantoea agglomerans* strain CT2		*Curvularia* sp and *Fusarium oxysporum* var. radicis-lycopersici strain 22	
*Pantoea ananatis* strain CT10		*Pythium ultimum*, *Curvularia* sp and *Fusarium oxysporum* var. radicis-lycopersici strain 27	
*Paenibacillus* sp. CT14		*Curvularia* sp	
*Microbacterium* sp. CT28		*Pythium ultimum*	
*Curtobacterium* sp.CT30		*Pythium ultimum*	
*Paenibacillus kribbensis* strain HS-R01	Rice cultivars (*Oryza sativa* var. Japonica c.v. Chilbo, Chuchung, Haiami, Ilpum	*Fusarium oxysporum* and *Rhizoctonia solani*	[83]
*Paenibacillus kribbensis* strain HS-R14		*Fusarium oxysporum* and *Rhizoctonia solani*	
*Bacillus aryabhattai* strain HS-S05		*Fusarium oxysporum*	
*Bacillus megaterium* strain KW7-R08		*Fusarium oxysporum*	
*Klebsiella pneumoniae* strain KW7-S06		*Fusarium oxysporum* and *Rhizoctonia solani*	
*Klebsiella pneumoniae* strain KW7-S22		*Fusarium oxysporum* and *Rhizoctonia solani*	
*Klebsiella pneumoniae* strain KW7-S27		*Fusarium oxysporum* and *Rhizoctonia solani*	
*Klebsiella pneumoniae* strain KW7-S33		*Fusarium oxysporum* and *Rhizoctonia solani*	
*Bacillus subtilis* strain CB-R05		*Fusarium oxysporum* and *Rhizoctonia solani*	
*Microbacterium binotii* strain CB-S18		*Fusarium oxysporum* and *Rhizoctonia solani*	
*Microbacterium trichotecenolyticum* strain SW521-L21		*Fusarium oxysporum* and *Rhizoctonia solani*	
*Microbacterium trichotecenolyticum* strain SW521-L37		*Fusarium oxysporum* and *Rhizoctonia solani*	

## Data Availability

Data is contained within the article or supplementary material.

## Supplementary Materials

The following are available online at https://www.mdpi.com/article/10.3390/ijms221810165/s1, Figure S1: Relative abundance of rice endophytic bacterial.

## References

[1] Malfanova N.V. (2013). Endophytic Bacteria with Plant Growth Promoting and Biocontrol Abilities. Ph.D. Thesis.

[2] Mundt J.O., Hinkle N.F. (1976). Bacteria within ovules and seeds. Appl. Environ. Microbiol..

[3] Sessitsch A., Hardoim P., Döring J., Weilharter A., Krause A., Woyke T., Mitter B., Hauberg-Lotte L., Friedrich F., Rahalkar M. (2012). Functional characteristics of an endophyte community colonizing rice roots as revealed by metagenomic analysis. Mol. Plant-Microbe Interact..

[4] Krause A., Julich H., Mankar M., Reinhold-Hurek B. (2017). The Regulatory Network controlling ethanol-induced expression of alcohol dehydrogenase in the endophyte *Azoarcus* sp. Strain BH72. Mol. Plant-Microbe Interact..

[5] Ahmad Z., Wu J., Chen L., Dong W. (2017). Isolated Bacillus subtilis strain 330-2 and its antagonistic genes identified by the removing PCR. Sci. Rep..

[6] Ludueña L.M., Anzuay M.S., Angelini J.G., McIntosh M., Becker A., Rupp O., Goesmann A., Blom J., Fabra A., Taurian T.J.G. (2019). Genome sequence of the endophytic strain *Enterobacter* sp. J49, a potential biofertilizer for peanut and maize. Genomics.

[7] Kandel S.L., Joubert P.M., Doty S.L. (2017). Bacterial Endophyte colonization and distribution within plants. Microorganisms.

[8] Andreozzi A., Prieto P., Mercado-Blanco J., Monaco S., Zampieri E., Romano S., Valè G., Defez R., Bianco C.J.E.M. (2019). Efficient colonization of the endophytes Herbaspirillum huttiense RCA24 and Enterobacter cloacae RCA25 influences the physiological parameters of *Oryza sativa* L. cv. Baldo rice. Environ. Microbiol..

[9] Tavares M.J., Nascimento F.X., Glick B.R., Rossi M.J. (2018). The expression of an exogenous ACC deaminase by the endophyte Serratia grimesii BXF1 promotes the early nodulation and growth of common bean. Lett. Appl. Microbiol..

[10] Passari A., Upadhyaya K., Singh G., Abdel-Azeem A., Thankappan S., Uthandi S., Hashem A., Allah E.F.A., Malik J.A., As A. (2019). Enhancement of disease resistance, growth potential, and photosynthesis in tomato (*Solanum lycopersicum*) by inoculation with an endophytic actinobacterium, *Streptomyces thermocarboxydus* strain BPSAC147. PLoS ONE.

[11] Gyaneshwar P., James E., Mathan N., Reddy P.M., Reinhold-Hurek B., Ladha J.K. (2001). Endophytic Colonization of Rice by a Diazotrophic Strain of *Serratia marcescens*. J. Bacteriol..

[12] Elferink M., Schierhorn F. (2016). Global demand for food is rising. Can we meet it. Harv. Bus. Rev..

[13] Vollset S.E., Goren E., Yuan C.-W., Cao J., E Smith A., Hsiao T., Bisignano C., Azhar G.S., Castro E., Chalek J. (2020). Fertility, mortality, migration, and population scenarios for 195 countries and territories from 2017 to 2100: A forecasting analysis for the Global Burden of Disease Study. Lancet.

[14] Directive 2009/128/EC R.E. (2009). Directive 2009/128/EC of the European Parliament and of the Council of 21 October 2009 establishing a framework for Community action to achieve the sustainable use of pesticides. Off. J. Eur. Union.

[15] European Union Pesticides Database Pesticides Database. http://ec.europa.eu/food/plant/protection/evaluation/database_act_subs_en.htm.

[16] Fang L. Overview of Biofertilizer Registration in China. https://agrochemical.chemlinked.com/chempedia/overview-biofertilizer-registration-china.

[17] Montesinos E. (2003). Development, registration and commercialization of microbial pesticides for plant protection. Int. Microbiol..

[18] Glick B.R., Karaturovic D.M., Newell P.C. (1995). A novel procedure for rapid isolation of plant growth promoting pseudomonads. Can. J. Microbiol..

[19] Khare E., Mishra J., Arora N.K. (2018). Multifaceted interactions between endophytes and plant: Developments and prospects. Front. Microbiol..

[20] Glick B.R. (2015). Beneficial Plant-Bacterial Interactions.

[21] Van Rossum G., Drake F.L. (2009). PYTHON 2.6 Reference Manual.

[22] Letunic I., Bork P.J.N.A.R. (2019). Interactive tree of life (iTOL) v4: Recent updates and new developments. Nucleic Acids Res..

[23] Kaga H., Mano H., Tanaka F., Watanabe A., Kaneko S., Morisaki H. (2009). Rice seeds as sources of endophytic bacteria. Microbes Environ..

[24] Shahzad R., Waqas M., Khan A.L., Asaf S., Khan M.A., Kang S.-M., Yun B.-W., Lee I.-J. (2016). Seed-borne endophytic Bacillus amyloliquefaciens RWL-1 produces gibberellins and regulates endogenous phytohormones of *Oryza sativa*. Plant Physiol. Biochem..

[25] Khaskheli M.A., Wu L., Chen G., Chen L., Hussain S., Song D., Liu S., Feng G. (2020). Isolation and Characterization of Root-Associated Bacterial Endophytes and Their Biocontrol Potential against Major Fungal Phytopathogens of Rice (*Oryza sativa* L.). Pathogens.

[26] Valdez-Nuñez R., Ríos-Ruiz W., Ormeño-Orrillo E., Torres-Chávez E., Torres-Delgado J. (2020). Genetic characterization of rice endophytic bacteria (*Oryza sativa* L.) with antimicrobial activity against Burkholderia glumae. Rev. Argent. Microbiol..

[27] Nasrollahi M., Pourbabaei A.A., Etesami H., Talebi K. (2020). Diazinon degradation by bacterial endophytes in rice plant (*Oryzia sativa* L.): A possible reason for reducing the efficiency of diazinon in the control of the rice stem–borer. Chemosphere.

[28] Defez R., Andreozzi A., Bianco C. (2017). The overproduction of indole-3-acetic acid (IAA) in endophytes upregulates nitrogen fixation in both bacterial cultures and inoculated rice plants. Microb. Ecol..

[29] Zhao J., Zhao X., Wang J., Gong Q., Zhang X., Zhang G. (2020). Isolation, Identification and Characterization of Endophytic Bacterium *Rhizobium oryzihabitans* sp. nov., from Rice Root with Biotechnological potential in agriculture. Microorganisms.

[30] Banik A., Mukhopadhaya S.K., Dangar T.K. (2016). Characterization of N 2-fixing plant growth promoting endophytic and epiphytic bacterial community of Indian cultivated and wild rice (*Oryza* spp.) genotypes. Planta.

[31] Zhao J.-J., Zhang J., Sun L., Zhang R.-J., Zhang C.-W., Yin H.-Q., Zhang X.-X. (2017). *Rhizobium oryziradicis* sp. nov., isolated from rice roots. Int. J. Syst. Evol. Microbiol..

[32] Kwak M.-J., Song J.Y., Kim S.-Y., Jeong H., Kang S.G., Kim B.K., Kwon S.-K., Lee C.H., Yu D.S., Park S.-H. (2012). Complete Genome Sequence of the Endophytic Bacterium *Burkholderia* sp. Strain KJ006. Am. Soc. Microbiol..

[33] Raj G., Shadab M., Deka S., Das M., Baruah J., Bharali R., Talukdar N.C. (2019). Seed interior microbiome of rice genotypes indigenous to three agroecosystems of Indo-Burma biodiversity hotspot. BMC Genom..

[34] Liu H., Zhang L., Meng A., Zhang J., Xie M., Qin Y., Faulk D.C., Zhang B., Yang S., Qiu L. (2017). Isolation and molecular identification of endophytic diazotrophs from seeds and stems of three cereal crops. PLoS ONE.

[35] Aljuraifani A., Aldosary S., Ababutain I. (2019). In vitro antimicrobial activity of endophytes, isolated from Moringa peregrina growing in eastern region of Saudi Arabia. Natl. Acad. Sci. Lett..

[36] Correa-Galeote D., Bedmar E.J., Arone G. (2018). Maize endophytic bacterial diversity as affected by soil cultivation history. Front. Microbiol..

[37] Marag P.S., Suman A. (2018). Growth stage and tissue specific colonization of endophytic bacteria having plant growth promoting traits in hybrid and composite maize (*Zea mays* L.). Microbiol. Res..

[38] Rania A.B.A., Jabnoun-Khiareddine H., Nefzi A., Mokni-Tlili S., Daami-Remadi M. (2016). Endophytic bacteria from Datura metel for plant growth promotion and bioprotection against Fusarium wilt in tomato. Biocontrol Sci. Technol..

[39] Zhang Q., Li Y., Xu F., Zheng M., Xi X., Zhang X., Han C. (2017). Optimization of submerged fermentation medium for matrine production by Aspergillus terreus, an endophytic fungus harboring seeds of Sophora flavescens, using response surface methodology. Mycobiology.

[40] Proença D.N., Francisco R., Kublik S., Schöler A., Vestergaard G., Schloter M., Morais P.V. (2017). The microbiome of endophytic, wood colonizing bacteria from pine trees as affected by pine wilt disease. Sci. Rep..

[41] Pereira G.V.D.M., Magalhães K.T., Lorenzetii E.R., Souza T.P., Schwan R.F. (2011). A Multiphasic approach for the identification of endophytic bacterial in strawberry fruit and their potential for plant growth promotion. Microb. Ecol..

[42] Kukkurainen S., Leino A., Vähämiko S., Kärkkäinen H.R., Ahanen K., Sorvari S., Rugienius R., Toldi O. (2005). Occurrence and location of endophytic bacteria in garden and wild strawberry. HortScience.

[43] Villalobos S.D.L.S., Robles R.I., Cota F.I.P., Larsen J., Lozano P., Tiedje J.M. (2019). *Bacillus cabrialesii* sp. nov., an endophytic plant growth promoting bacterium isolated from wheat (*Triticum turgidum* subsp. durum) in the Yaqui Valley, Mexico. Int. J. Syst. Evol. Microbiol..

[44] Paz I.C.P., Santin R.C.M., Guimaraes A.M., Rosa O.P.P., Dias A.C.F., Quecine M., Azevedo J., Matsumura A.T.S. (2012). Eucalyptus growth promotion by endophytic *Bacillus* spp.. Genet. Mol. Res..

[45] Hazarika D.J., Goswami G., Gautom T., Parveen A., Das P., Barooah M., Boro R.C. (2019). Lipopeptide mediated biocontrol activity of endophytic Bacillus subtilis against fungal phytopathogens. BMC Microbiol..

[46] Bruisson S., Zufferey M., L’Haridon F., Trutmann E., Anand A., Dutartre A., De Vrieze M., Weisskopf L. (2019). Endophytes and epiphytes from the grapevine leaf microbiome as potential biocontrol agents against phytopathogens. Front. Microbiol..

[47] Bell C., Dickie G.A., Harvey W., Chan J. (2011). Endophytic bacteria in grapevine. Can. J. Microbiol..

[48] Schmidt C., Lovecká P., Mrnka L., Vychodilová A., Strejček M., Fenclová M., Demnerová K.J.M.E. (2018). Distinct communities of poplar endophytes on an unpolluted and a risk element-polluted site and their plant growth-promoting potential in vitro. Microb. Ecol..

[49] Sziderics A.H., Rasche F., Trognitz F., Sessitsch A., Wilhelm E. (2007). Bacterial endophytes contribute to abiotic stress adaptation in pepper plants (*Capsicum annuum* L.). Can. J. Microbiol..

[50] Tuo L., Yan X.R., Li F.N., Bao Y.X., Shi H.C., Li H.Y., Sun C.H. (2018). *Brachybacterium endophyticum* sp. nov., a novel endophytic actinobacterium isolated from bark of *Scutellaria baicalensis* Georgi. Int. J. Syst. Evol. Microbiol..

[51] Shaikh A., Parmar P., Katagi R., Patel D., Desai H.R., Solanki B.G. (2017). Bioprospecting Potential of Endophytic Bacteria from Leaves of *Gossypium hirsutum*. Int. J. Curr. Microbiol. Appl. Sci..

[52] Boiu-sicuia O.-A., Constantinescu F., Cornea C.P.J.S.B. (2017). Selection and characterization of new endophytic bacterial strains isolated from potato tuber useful in biocontrol strategies. Sci. Bull..

[53] Mahmood A., Takagi K., Ito K., Kataoka R. (2019). Changes in endophytic bacterial communities during different growth stages of cucumber (*Cucumis sativus* L.). World J. Microbiol. Biotechnol..

[54] Abdallah R.A.B., Mokni-Tlili S., Nefzi A., Jabnoun-Khiareddine H., Daami-Remadi M. (2016). Biocontrol of Fusarium wilt and growth promotion of tomato plants using endophytic bacteria isolated from *Nicotiana glauca* organs. Biol. Control.

[55] Wang X., Liang G. (2014). Control efficacy of an endophytic Bacillus amyloliquefaciens strain BZ6-1 against peanut bacterial wilt, *Ralstonia solanacearum*. BioMed Res. Int..

[56] Murugappan R., Begum S.B., Roobia R.R. (2013). Symbiotic influence of endophytic Bacillus pumilus on growth promotion and probiotic potential of the medicinal plant *Ocimum sanctum*. Symbiosis.

[57] Tariq M., Hameed S., Yasmeen T., Zahid M., Zafar M. (2014). Molecular characterization and identification of plant growth promoting endophytic bacteria isolated from the root nodules of pea (*Pisum sativum* L.). World J. Microbiol. Biotechnol..

[58] Molina M.A., Ramos J.L., Espinosa-Urgel M.J.E.M. (2006). A two-partner secretion system is involved in seed and root colonization and iron uptake by Pseudomonas putida KT2440. Environ. Microbiol..

[59] Borah M., Das S., Boruah H., Boro R., Barooah M.J.B. (2018). Diversity of culturable endophytic bacteria from wild and cultivated rice showed potential plant growth promoting activities. bioRxiv.

[60] Zhou J., Li P., Meng D., Gu Y., Zheng Z., Yin H., Zhou Q., Li J. (2020). Isolation, characterization and inoculation of Cd tolerant rice endophytes and their impacts on rice under Cd contaminated environment. Environ. Pollut..

[61] Walitang D.I., Kim C.G., Jeon S., Kang Y., Sa T. (2019). Conservation and transmission of seed bacterial endophytes across generations following crossbreeding and repeated inbreeding of rice at different geographic locations. MicrobiologyOpen.

[62] Hardoim P., Hardoim C., Van Overbeek L.S., Van Elsas J.D. (2012). Dynamics of Seed-Borne Rice Endophytes on Early Plant Growth Stages. PLoS ONE.

[63] Mitter B., Pfaffenbichler N., Flavell R., Compant S., Antonielli L., Petric A., Berninger T., Naveed M., Sheibani-Tezerji R., von Maltzahn G. (2017). A New Approach to modify plant microbiomes and traits by introducing beneficial bacteria at flowering into progeny seeds. Front. Microbiol..

[64] Manirajan B.A., Ratering S., Rusch V., Schwiertz A., Geissler-Plaum R., Cardinale M., Schnell S. (2016). Bacterial microbiota associated with flower pollen is influenced by pollination type, and shows a high degree of diversity and species-specificity. Environ. Microbiol..

[65] Agarwal V.K., Sinclair J.B. (1996). Principles of Seed Pathology.

[66] Pfeiffer B., Fender A.-C., Lasota S., Hertel D., Jungkunst H.F., Daniel R. (2013). Leaf litter is the main driver for changes in bacterial community structures in the rhizosphere of ash and beech. Appl. Soil Ecol..

[67] Breidenbach B., Pump J., Dumont M.G. (2016). Microbial community structure in the rhizosphere of rice plants. Front. Microbiol..

[68] Berendsen R.L., Pieterse C.M., Bakker P.A. (2012). The rhizosphere microbiome and plant health. Trends Plant Sci..

[69] Liu H., Carvalhais L.C., Crawford M., Singh E., Dennis P.G., Pieterse C., Schenk P.M. (2017). Inner plant values: Diversity, colonization and benefits from endophytic bacteria. Front. Microbiol..

[70] Okunishi S., Sako K., Mano H., Imamura A., Morisaki H. (2005). Bacterial flora of endophytes in the maturing seed of cultivated rice (*Oryza sativa*). Microbes Environ..

[71] Durán P., Acuña J.J., Tapia M., Azcón R., Paredes C., Rengel Z., Mora M.L. (2014). Endophytic bacteria from selenium-supplemented wheat plants could be useful for plant-growth promotion, biofortification and Gaeumannomyces graminis biocontrol in wheat production. Biol. Fertil. Soils.

[72] Ren J.-H., Li H., Wang Y.-F., Ye J.-R., Yan A.-Q., Wu X.-Q. (2013). Biocontrol potential of an endophytic Bacillus pumilus JK-SX001 against poplar canker. Biol. Control.

[73] Mohamad O.A., Li L., Ma J.-B., Hatab S., Xu L., Guo J.-W., Rasulov B.A., Liu Y.-H., Hedlund B.P., Li W.-J. (2018). Evaluation of the antimicrobial activity of endophytic bacterial populations from Chinese traditional medicinal plant licorice and characterization of the bioactive secondary metabolites produced by Bacillus atrophaeus against Verticillium dahliae. Front. Microbiol..

[74] Shi Y., Yang H., Zhang T., Sun J., Lou K. (2014). Illumina-based analysis of endophytic bacterial diversity and space-time dynamics in sugar beet on the north slope of Tianshan mountain. Appl. Microbiol. Biotechnol..

[75] Elbeltagy A., Nishioka K., Suzuki H., Sato T., Sato Y.-I., Morisaki H., Mitsui H., Minamisawa K. (2000). Isolation and characterization of endophytic bacteria from wild and traditionally cultivated rice varieties. Soil Sci. Plant Nutr..

[76] Compant S., Van Der Heijden M.G.A., Sessitsch A. (2010). Climate change effects on beneficial plant–microorganism interactions. FEMS Microbiol. Ecol..

[77] Penuelas J., Rico L., Ogaya R., Jump A., Terradas J.J.P.B. (2012). Summer season and long-term drought increase the richness of bacteria and fungi in the foliar phyllosphere of Quercus ilex in a mixed Mediterranean forest. Plant Biol..

[78] Ren G., Zhang H., Lin X., Zhu J., Jia Z. (2015). Response of leaf endophytic bacterial community to elevated CO_2_ at different growth stages of rice plant. Front. Microbiol..

[79] Walitang D.I., Kim C.-G., Kim K., Kang Y., Kim Y.K., Sa T. (2018). The influence of host genotype and salt stress on the seed endophytic community of salt-sensitive and salt-tolerant rice cultivars. BMC Plant Biol..

[80] Bogas A.C., Ferreira A.J., Araújo W.L., Astolfi-Filho S., Kitajima E.W., Lacava P.T., Azevedo J.L. (2015). Endophytic bacterial diversity in the phyllosphere of Amazon Paullinia cupana associated with asymptomatic and symptomatic anthracnose. Springerplus.

[81] Singh R.K., Mishra R.P.N., Jaiswal H.K., Kumar V., Pandey S.P., Rao S.B., Annapurna K. (2006). Isolation and Identification of Natural Endophytic Rhizobia from Rice (*Oryza sativa* L.) Through rDNA PCR-RFLP and Sequence Analysis. Curr. Microbiol..

[82] Mano H., Tanaka F., Watanabe A., Kaga H., Okunishi S., Morisaki H. (2006). Culturable surface and endophytic bacterial flora of the maturing seeds of rice plants (*Oryza sativa*) cultivated in a paddy field. Microbes Environ..

[83] Ji S.H., Gururani M., Chun S.-C. (2013). Isolation and characterization of plant growth promoting endophytic diazotrophic bacteria from Korean rice cultivars. Microbiol. Res..

[84] Ferrando L., Mañay J.F., Scavino A.F. (2012). Molecular and culture-dependent analyses revealed similarities in the endophytic bacterial community composition of leaves from three rice (*Oryza sativa*) varieties. FEMS Microbiol. Ecol..

[85] Yang H., Sun X., Song W., Wang Y., Cai M. (1999). Screening, identification and distribution of endophytic associative diazotrophs isolated from rice plants. Acta Bot. Sin..

[86] Zhang C.-W., Zhang J., Zhao J.-J., Zhao X., Zhao D.-F., Yin H.-Q., Zhang X.-X. (2017). *Serratia oryzae* sp. nov., isolated from rice stems. Int. J. Syst. Evol. Microbiol..

[87] Xiong X.Q., Liao H.D., Ma J.S., Liu X., Zhang L., Shi X., Yang X., Lu X., Zhu Y. (2014). Isolation of a rice endophytic bacterium, *P antoea* sp. S d-1, with ligninolytic activity and characterization of its rice straw degradation ability. Lett. Appl. Microbiol..

[88] Wang X., He S.-W., Guo H.-B., Thin K.K., Gao J.-S., Wang Y., Zhang X.-X. (2020). *Pseudomonas rhizoryzae* sp. nov., isolated from rice. Int. J. Syst. Evol. Microbiol..

[89] Mosquito S., Bertani I., Licastro D., Compant S., Myers M.P., Hinarejos E., Levy A., Venturi V. (2020). In planta colonization and role of T6SS in two rice Kosakonia endophytes. Mol. Plant-Microbe Interact..

[90] Eevers N., Gielen M., Sánchez-López A., Jaspers S., White J.C., Vangronsveld J., Weyens N. (2015). Optimization of isolation and cultivation of bacterial endophytes through addition of plant extract to nutrient media. Microb. Biotechnol..

[91] Tholozan J., Cappelier J., Tissier J., Delattre G., Federighi M.J.A., Microbiology E. (1999). Physiological Characterization of Viable-but-Nonculturable Campylobacter Jejuni cells. Appl. Environ. Microbiol..

[92] Ma Y., Prasad M., Rajkumar M., Freitas H. (2011). Plant growth promoting rhizobacteria and endophytes accelerate phytoremediation of metalliferous soils. Biotechnol. Adv..

[93] Schleifer K.H. (2009). Classification of Bacteria and Archaea: Past, present and future. Syst. Appl. Microbiol..

[94] Girard G., Traag B.A., Sangal V., Mascini N., Hoskisson P., Goodfellow M., van Wezel G.P. (2013). A novel taxonomic marker that discriminates between morphologically complex actinomycetes. Open Biol..

[95] Ventura M., Canchaya C., Tauch A., Chandra G., Fitzgerald G.F., Chater K.F., van Sinderen D. (2007). Genomics of Actinobacteria: Tracing the Evolutionary History of an Ancient Phylum. Microbiol. Mol. Biol. Rev..

[96] Nouioui I., Carro L., García-López M., Meier-Kolthoff J.P., Woyke T., Kyrpides N., Pukall R., Klenk H.-P., Goodfellow M., Göker M. (2018). Genome-based taxonomic classification of the phylum actinobacteria. Front. Microbiol..

[97] Goris J., Konstantinidis K.T., Klappenbach J.A., Coenye T., Vandamme P., Tiedje J.M. (2007). DNA–DNA hybridization values and their relationship to whole-genome sequence similarities. Int. J. Syst. Evol. Microbiol..

[98] Maiden M.C. (2006). Multilocus Sequence Typing of Bacteria. Annu. Rev. Microbiol..

[99] Saito A., Ikeda S., Ezura H., Minamisawa K. (2007). Microbial Community Analysis of the Phytosphere Using Culture-Independent Methodologies. Microbes Environ..

[100] Fisher M.M., Triplett E.W. (1999). Automated Approach for Ribosomal Intergenic Spacer Analysis of Microbial Diversity and Its Application to Freshwater Bacterial Communities. Appl. Environ. Microbiol..

[101] Ma Y., Rajkumar M., Zhang C., Freitas H. (2016). Beneficial role of bacterial endophytes in heavy metal phytoremediation. J. Environ. Manag..

[102] Reiter B., Pfeifer U., Schwab H., Sessitsch A. (2002). Response of Endophytic Bacterial Communities in Potato Plants to Infection with Erwinia carotovora subsp. atroseptica. Appl. Environ. Microbiol..

[103] Thijs S., van Dillewijn P., Sillen W., Truyens S., Holtappels M., D’haen J., Carleer R., Weyens N., Ameloot M., Ramos J.L. (2014). Exploring the rhizospheric and endophytic bacterial communities of Acer pseudoplatanus growing on a TNT-contaminated soil: Towards the development of a rhizocompetent TNT-detoxifying plant growth promoting consortium. Plant Soil.

[104] Annapurna K., Govindasamy V., Sharma M., Ghosh A., Chikara S.K. (2018). Whole genome shotgun sequence of Bacillus paralicheniformis strain KMS 80, a rhizobacterial endophyte isolated from rice (*Oryza sativa* L.). 3 Biotech.

[105] Tian X., Cao L., Tan H., Han W.-Q., Chen M., Liu Y., Zhou S. (2007). Diversity of Cultivated and Uncultivated Actinobacterial Endophytes in the Stems and Roots of Rice. Microb. Ecol..

[106] Ren G., Zhu C., Alam M.S., Tokida T., Sakai H., Nakamura H., Usui Y., Zhu J., Hasegawa T., Jia Z. (2015). Response of soil, leaf endosphere and phyllosphere bacterial communities to elevated CO_2_ and soil temperature in a rice paddy. Plant Soil.

[107] Battu L., Reddy M.M., Goud B.S., Ulaganathan K., Kandasamy U. (2017). Genome inside genome: NGS based identification and assembly of endophytic Sphingopyxis granuli and Pseudomonas aeruginosa genomes from rice genomic reads. Genomics.

[108] Manter D.K., Delgado J.A., Holm D.G., Stong R.A. (2010). Pyrosequencing reveals a highly diverse and cultivar-specific bacterial endophyte community in potato roots. Microb. Ecol..

[109] He Y., Zhou B.-J., Deng G.-H., Jiang X.-T., Zhang H., Zhou H.-W. (2013). Comparison of microbial diversity determined with the same variable tag sequence extracted from two different PCR amplicons. BMC Microbiol..

[110] Akinsanya M., Goh J.K., Lim S.P., Ting A.S.Y. (2015). Metagenomics study of endophytic bacteria in Aloe vera using next-generation technology. Genom. Data.

[111] Ma Y., Oliveira R.S., Nai F., Rajkumar M., Luo Y., Rocha I.D.S., Freitas H. (2015). The hyperaccumulator Sedum plumbizincicola harbors metal-resistant endophytic bacteria that improve its phytoextraction capacity in multi-metal contaminated soil. J. Environ. Manag..

[112] Miliute I., Buzaite O., Baniulis D., Stanys V. (2015). Bacterial endophytes in agricultural crops and their role in stress tolerance: A review. Zemdirb. Agric..

[113] Ivleva N.B., Groat J., Staub J.M., Stephens M. (2016). Expression of Active Subunit of Nitrogenase via Integration into Plant Organelle Genome. PLoS ONE.

[114] Bhattacharjee R.B., Singh A., Mukhopadhyay S.N. (2008). Use of nitrogen-fixing bacteria as biofertiliser for non-legumes: Prospects and challenges. Appl. Microbiol. Biotechnol..

[115] Kim J., Rees D.C. (1994). Nitrogenase and biological nitrogen fixation. Biochemistry.

[116] Pallai R. (2005). Effect of Plant Growth-Promoting Rhizobacteria on Canola (*Brassica napus* L) and Lentil (Lens culinaris Medik) Plants.

[117] Ladha J., De Bruijn F., Malik K. (1997). Introduction: Assessing opportunities for nitrogen fixation in rice-a frontier project. Plant Soil.

[118] Holguin G., Patten C.L. (1999). Biochemical and Genetic Mechanisms Used by Plant Growth Promoting Bacteria.

[119] Wang L., Zhang L., Liu Z., Zhao D., Liu X., Zhang B., Xie J., Hong Y., Li P., Chen S. (2013). A minimal nitrogen fixation gene cluster from *Paenibacillus* sp. WLY78 enables expression of active nitrogenase in *Escherichia coli*. PLoS Genet..

[120] Bishop P.E., Joerger R.D. (1990). Genetics and molecular biology of alternative nitrogen fixation systems. Annu. Rev. Plant Biol..

[121] Cocking E.C. (2003). Endophytic colonization of plant roots by nitrogen-fixing bacteria. Plant Soil.

[122] Ding T., Su B., Chen X., Xie S., Gu S., Wang Q., Huang D., Jiang H. (2017). An endophytic bacterial strain isolated from Eucommia ulmoides inhibits southern corn leaf blight. Front. Microbiol..

[123] Kandel S.L., Firrincieli A., Joubert P.M., Okubara P.A., Leston N.D., McGeorge K.M., Mugnozza G.S., Harfouche A., Kim S.-H., Doty S.L. (2017). An in vitro study of bio-control and plant growth promotion potential of Salicaceae endophytes. Front. Microbiol..

[124] Amaral F.P.D., Bueno J.C.F., Hermes V.S., Arisi A.C.M. (2014). Gene expression analysis of maize seedlings (DKB240 variety) inoculated with plant growth promoting bacterium Herbaspirillum seropedicae. Symbiosis.

[125] Carrell A.A., Frank A.C. (2014). Pinus flexilis and Picea engelmannii share a simple and consistent needle endophyte microbiota with a potential role in nitrogen fixation. Front. Microbiol..

[126] Ahemad M. (2014). Phosphate-solubilizing bacteria-assisted phytoremediation of metalliferous soils: A review. 3 Biotech.

[127] Afzal I., Shinwari Z.K., Sikandar S., Shahzad S. (2019). Plant beneficial endophytic bacteria: Mechanisms, diversity, host range and genetic determinants. Microbiol. Res..

[128] Maheshwari R., Bhutani N., Suneja P. (2019). Screening and characterization of siderophore producing endophytic bacteria from Cicer arietinum and Pisum sativum plants. J. Appl. Biol. Biotechnol..

[129] Abedinzadeh M., Etesami H., Alikhani H.A. (2019). Characterization of rhizosphere and endophytic bacteria from roots of maize (*Zea mays* L.) plant irrigated with wastewater with biotechnological potential in agriculture. Biotechnol. Rep..

[130] Wang S., Wang W., Jin Z., Du B., Ding Y., Ni T., Jiao F. (2013). Screening and diversity of plant growth promoting endophytic bacteria from peanut. Afr. J. Microbiol. Res..

[131] Gharu A., Tarafdar J. (2004). Influence of organic acids on mobilization of inorganic and organic phosphorus in soil. J. Indian Soc. Soil Sci..

[132] Chen Y., Rekha P., Arun A., Shen F., Lai W.-A., Young C. (2006). Phosphate solubilizing bacteria from subtropical soil and their tricalcium phosphate solubilizing abilities. Appl. Soil Ecol..

[133] Khan K.S., Joergensen R.G. (2009). Changes in microbial biomass and P fractions in biogenic household waste compost amended with inorganic P fertilizers. Bioresour. Technol..

[134] Palaniappan P., Chauhan P.S., Saravanan V., Anandham R., Sa T. (2010). Isolation and characterization of plant growth promoting endophytic bacterial isolates from root nodule of *Lespedeza* sp.. Biol. Fertil. Soils.

[135] Puente M.E., Li C.Y., Bashan Y. (2009). Rock-degrading endophytic bacteria in cacti. Environ. Exp. Bot..

[136] Goswami D., Vaghela H., Parmar S., Dhandhukia P., Thakker J.N. (2013). Plant growth promoting potentials of Pseudomonas spp. strain OG isolated from marine water. J. Plant Interact..

[137] Patel K., Goswami D., Dhandhukia P., Thakker J., Maheshwari D. (2015). Techniques to study microbial phytohormones. Bacterial Metabolites in Sustainable Agroecosystem. Sustainable Development and Biodiversity.

[138] Khan M.M.A., Haque E., Paul N.C., Khaleque M.A., Al-Garni S.M., Rahman M., Islam M.T. (2017). Enhancement of growth and grain yield of rice in nutrient deficient soils by rice probiotic bacteria. Rice Sci..

[139] Phetcharat P., Duangpaeng A. (2012). Screening of endophytic bacteria from organic rice tissue for indole acetic acid production. Procedia Eng..

[140] Xu T., Cao L., Zeng J., Franco C., Yang Y., Hu X., Liu Y., Wang X., Gao Y., Bu Z. (2019). The antifungal action mode of the rice endophyte Streptomyces hygroscopicus OsiSh-2 as a potential biocontrol agent against the rice blast pathogen. Pestic. Biochem. Physiol..

[141] Spaepen S., Vanderleyden J. (2011). Auxin and plant-microbe interactions. Cold Spring Harb. Perspect. Biol..

[142] Glick B.R. (2012). Plant Growth-Promoting Bacteria: Mechanisms and Applications. Scientifica.

[143] Mole B.M., Baltrus D.A., Dangl J.L., Grant S.R. (2007). Global virulence regulation networks in phytopathogenic bacteria. Trends Microbiol..

[144] Spaepen S., Vanderleyden J., Remans R. (2007). Indole-3-acetic acid in microbial and microorganism-plant signaling. FEMS Microbiol. Rev..

[145] Xu T., Li Y., Zeng X., Yang X., Yang Y., Yuan S., Hu X., Zeng J., Wang Z., Liu Q. (2017). Isolation and evaluation of endophytic Streptomyces endus OsiSh-2 with potential application for biocontrol of rice blast disease. J. Sci. Food Agric..

[146] Leveau J.H.J., Lindow S.E. (2005). Utilization of the Plant Hormone Indole-3-Acetic Acid for Growth by Pseudomonas putida Strain 1290. Appl. Environ. Microbiol..

[147] Asaf S., Khan M.A., Khan A.L., Waqas M., Shahzad R., Kim A.-Y., Kang S.-M., Lee I.-J. (2017). Bacterial endophytes from arid land plants regulate endogenous hormone content and promote growth in crop plants: An example of *Sphingomonas* sp. and *Serratia marcescens*. J. Plant Interact..

[148] Herrera S.D., Grossi C., Zawoznik M., Groppa M.D. (2016). Wheat seeds harbour bacterial endophytes with potential as plant growth promoters and biocontrol agents of *Fusarium graminearum*. Microbiol. Res..

[149] Ortiz-Castro R., Contreras-Cornejo H.A., Macías-Rodríguez L., López-Bucio J. (2009). The role of microbial signals in plant growth and development. Plant Signal. Behav..

[150] Persello-Cartieaux F., Nussaume L., Robaglia C. (2003). Tales from the underground: Molecular plant–rhizobacteria interactions. Plant Cell Environ..

[151] Nieto K., Frankenberger W. (1990). Influence of adenine, isopentyl alcohol and *Azotobacter chroococcum* on the growth of *Raphanus sativus*. Plant Soil.

[152] Da K., Nowak J., Flinn B. (2012). Potato cytosine methylation and gene expression changes induced by a beneficial bacterial endophyte, *Burkholderia phytofirmans* strain PsJN. Plant Physiol. Biochem..

[153] Bhore S.J., Ravichantar N., Loh C.Y. (2010). Screening of endophytic bacteria isolated from leaves of Sambung Nyawa [*Gynura procumbens* (Lour.) Merr.] for cytokinin-like compounds. Bioinformation.

[154] Dalal J., Kulkarni N. (2014). Antagonistic and Plant Growth Promoting Potentials of Indigenous Endophytic Actinomycetes of Soybean (*Glycine max* (L) Merril). CIBTech J. Microbiol..

[155] Dodd I., Zinovkina N., Safronova V., Belimov A. (2010). Rhizobacterial mediation of plant hormone status. Ann. Appl. Biol..

[156] Tsavkelova E., Klimova S.Y., Cherdyntseva T., Netrusov A. (2006). Microbial producers of plant growth stimulators and their practical use: A review. Appl. Biochem. Microbiol..

[157] Kang S.-M., Joo G.-J., Hamayun M., Na C.-I., Shin D.-H., Kim H.Y., Hong J.-K., Lee I.-J. (2009). Gibberellin production and phosphate solubilization by newly isolated strain of Acinetobacter calcoaceticus and its effect on plant growth. Biotechnol. Lett..

[158] Ishak Z., Mohd Iswadi M., Russman Nizam A., Ahmad Kamil M., Ernie Eileen R., Wan Syaidatul A., Ainon H. (2016). Plant growth hormones produced by endophytic Bacillus subtilis strain LKM-BK isolated from cocoa. Malays. Cocoa J..

[159] Dugardeyn J., Van Der Straeten D. (2008). Ethylene: Fine-tuning plant growth and development by stimulation and inhibition of elongation. Plant Sci..

[160] Belimov A., Hontzeas N., Safronova V., Demchinskaya S., Piluzza G., Bullitta S., Glick B. (2005). Cadmium-tolerant plant growth-promoting bacteria associated with the roots of Indian mustard (*Brassica juncea* L. Czern.). Soil Biol. Biochem..

[161] Giovanelli J., Mudd S.H., Datko A.H. (1980). Sulfur amino acids in plants. Amino Acids and Derivatives.

[162] Glick B.R., Todorovic B., Czarny J., Cheng Z., Duan J., McConkey B. (2007). Promotion of plant growth by bacterial ACC deaminase. Crit. Rev. Plant Sci..

[163] Shaharoona B., Arshad M., Zahir Z. (2006). Effect of plant growth promoting rhizobacteria containing ACC-deaminase on maize (*Zea mays* L.) growth under axenic conditions and on nodulation in mung bean (*Vigna radiata* L.). Lett. Appl. Microbiol..

[164] Grichko V.P., Glick B.R. (2001). Amelioration of flooding stress by ACC deaminase-containingplant growth-promoting bacteria. Plant Physiol. Biochem..

[165] Mayak S., Tirosh T., Glick B.R. (2004). Plant growth-promoting bacteria that confer resistance to water stress in tomatoes and peppers. Plant Sci..

[166] Zahir Z.A., Ghani U., Naveed M., Nadeem S.M., Asghar H.N. (2009). Comparative effectiveness of Pseudomonas and Serratia sp. containing ACC-deaminase for improving growth and yield of wheat (*Triticum aestivum* L.) under salt-stressed conditions. Arch. Microbiol..

[167] Berg G. (2009). Plant–microbe interactions promoting plant growth and health: Perspectives for controlled use of microorganisms in agriculture. Appl. Microbiol. Biotechnol..

[168] Smyth E., McCarthy J., Nevin R., Khan M., Dow M., O’Gara F., Doohan F. (2011). In vitro analyses are not reliable predictors of the plant growth promotion capability of bacteria; a Pseudomonas fluorescens strain that promotes the growth and yield of wheat. J. Appl. Microbiol..

[169] Perrig D., Boiero M., Masciarelli O., Penna C., Ruiz O., Cassán F., Luna M. (2007). Plant-growth-promoting compounds produced by two agronomically important strains of Azospirillum brasilense, and implications for inoculant formulation. Appl. Microbiol. Biotechnol..

[170] Spaepen S., Das F., Luyten E., Michiels J., Vanderleyden J. (2009). Indole-3-acetic acid-regulated genes in Rhizobium etli CNPAF512. FEMS Microbiol. Lett..

[171] Malfanova N., Kamilova F., Validov S., Shcherbakov A., Chebotar V., Tikhonovich I., Lugtenberg B. (2011). Characterization of Bacillus subtilis HC8, a novel plant-beneficial endophytic strain from giant hogweed. Microb. Biotechnol..

[172] Melnick R.L., Zidack N.K., Bailey B.A., Maximova S.N., Guiltinan M., Backman P.A. (2008). Bacterial endophytes: *Bacillus* spp. from annual crops as potential biological control agents of black pod rot of cacao. Biol. Control.

[173] Liu B., Huang L., Buchenauer H., Kang Z. (2010). Isolation and partial characterization of an antifungal protein from the endophytic Bacillus subtilis strain EDR4. Pestic. Biochem. Physiol..

[174] Li H., Wang X., Han M., Zhao Z., Wang M., Tang Q., Liu C., Kemp B., Gu Y., Shuang J. (2012). Endophytic Bacillus subtilis ZZ120 and its potential application in control of replant diseases. Afr. J. Biotechnol..

[175] Villa-Rodríguez E., Parra-Cota F., Castro-Longoria E., López-Cervantes J., de los Santos-Villalobos S. (2019). Bacillus subtilis TE3: A promising biological control agent against Bipolaris sorokiniana, the causal agent of spot blotch in wheat (*Triticum turgidum* L. subsp. durum). Biol. Control.

[176] Rangjaroen C., Lumyong S., Sloan W.T., Sungthong R. (2019). Herbicide-tolerant endophytic bacteria of rice plants as the biopriming agents for fertility recovery and disease suppression of unhealthy rice seeds. BMC Plant Biol..

[177] Chung E.J., Hossain M.T., Khan A., Kim K.H., Jeon C.O., Chung Y.R. (2015). *Bacillus oryzicola* sp. nov., an endophytic bacterium isolated from the roots of rice with antimicrobial, plant growth promoting, and systemic resistance inducing activities in rice. Plant Pathol. J..

[178] Verma S.K., Kingsley K., Irizarry I., Bergen M., Kharwar R., White J.F. (2017). Seed-vectored endophytic bacteria modulate development of rice seedlings. J. Appl. Microbiol..

[179] Ruiza D., Agaras B.C., De Werrab P., Wall L.G., Valverde C. (2011). Characterization and screening of plant probiotic traits of bacteria isolated from rice seeds cultivated in Argentina. J. Microbiol..

[180] Santoyo G., Moreno-Hagelsieb G., Orozco-Mosqueda M.D.C., Glick B.R. (2016). Plant growth-promoting bacterial endophytes. Microbiol. Res..

[181] Van Rij E.T., Wesselink M., Chin-A-Woeng T.F., Bloemberg G.V., Lugtenberg B.J. (2004). Influence of environmental conditions on the production of phenazine-1-carboxamide by Pseudomonas chlororaphis PCL1391. Mol. Plant-Microbe Interact..

[182] Jousset A., Rochat L., Lanoue A., Bonkowski M., Keel C., Scheu S. (2011). Plants respond to pathogen infection by enhancing the antifungal gene expression of root-associated bacteria. Mol. Plant-Microbe Interact..

[183] Dekkers L.C., Mulders I.H.M., Phoelich C.C., Chin-A-Woeng T.F.C., Wijfjes A.H.M., Lugtenberg B.J.J. (2000). The sss Colonization Gene of the Tomato-Fusarium oxysporum f. sp. radicis-lycopersici Biocontrol strain pseudomonas fluorescens wcs365 can improve root colonization of other wild-type Pseudomonas spp. bacteria. Mol. Plant-Microbe Interactions.

[184] Haas D., Défago G. (2005). Biological control of soil-borne pathogens by fluorescent pseudomonads. Nat. Rev. Microbiol..

[185] Benizri E., Baudoin E., Guckert A. (2001). Root Colonization by inoculated plant growth-promoting rhizobacteria. Biocontrol Sci. Technol..

[186] Zhang L.H., Dong Y.H. (2004). Quorum sensing and signal interference: Diverse implications. Mol. Microbiol..

[187] Uroz S., Dessaux Y., Oger P. (2009). Quorum sensing and quorum quenching: The yin and yang of bacterial communication. ChemBioChem.

[188] Müller H., Westendorf C., Leitner E., Chernin L., Riedel K., Schmidt S., Eberl L., Berg G. (2009). Quorum-sensing effects in the antagonistic rhizosphere bacterium Serratia plymuthica HRO-C48. FEMS Microbiol. Ecol..

[189] Dandurishvili N., Toklikishvili N., Ovadis M., Eliashvili P., Giorgobiani N., Keshelava R., Tediashvili M., Vainstein A., Khmel I., Szegedi E. (2011). Broad-range antagonistic rhizobacteria Pseudomonas fluorescens and Serratia plymuthica suppress Agrobacterium crown gall tumours on tomato plants. J. Appl. Microbiol..

[190] Chernin L., Toklikishvili N., Ovadis M., Kim S., Ben-Ari J., Khmel I., Vainstein A. (2011). Quorum-sensing quenching by rhizobacterial volatiles. Environ. Microbiol. Rep..

[191] Berg G., Hallmann J. (2006). Control of plant pathogenic fungi with bacterial endophytes. Microbial Root Endophytes.

[192] El-Tarabily K.A. (2006). Rhizosphere-competent isolates of streptomycete and non-streptomycete actinomycetes capable of producing cell-wall-degrading enzymes to control Pythium aphanidermatum damping-off disease of cucumber. Botany.

[193] Ordentlich A., Elad Y., Chet I. (1988). The role of chitinase of Serratia marcescens in biocontrol of Sclerotium rolfsii. Phytopathology.

[194] Araújo W.L., Marcon J., Maccheroni W., van Elsas J.D., van Vuurde J.W., Azevedo J.L. (2002). Diversity of endophytic bacterial populations and their interaction with Xylella fastidiosa in citrus plants. Appl. Environ. Microbiol..

[195] Alvin A., Miller K.I., Neilan B.A. (2014). Exploring the potential of endophytes from medicinal plants as sources of antimycobacterial compounds. Microbiol. Res..

[196] Pieterse C.M., Van der Does D., Zamioudis C., Leon-Reyes A., Van Wees S.C. (2012). Hormonal modulation of plant immunity. Annu. Rev. Cell Dev. Biol..

[197] Akhtar M.S., Siddiqui Z.A. (2010). Role of plant growth promoting rhizobacteria in biocontrol of plant diseases and sustainable agriculture. Plant Growth and Health Promoting Bacteria.

[198] Kamilova F., Validov S., Azarova T., Mulders I., Lugtenberg B. (2005). Enrichment for enhanced competitive plant root tip colonizers selects for a new class of biocontrol bacteria. Environ. Microbiol..

[199] Van Loon L., Bakker P., Siddiqui Z.A. (2003). Induced systemic resistance as a mechanism of disease suppression by rhizobacteria. PGPR: Biocontrol and Biofertilization.

[200] Van Loon L., Bakker P., Gnanamanickam S.S. (2007). Root-associated bacteria inducing systemic resistance. Plant-Associated Bacteria.

[201] Bolyen E., Rideout J.R., Dillon M.R., Bokulich N.A., Abnet C.C., Al-Ghalith G.A., Alexander H., Alm E.J., Arumugam M., Asnicar F. (2019). Reproducible, interactive, scalable and extensible microbiome data science using QIIME 2. Nat. Biotechnol..

[202] Caporaso J.G., Bittinger K., Bushman F., DeSantis T.Z., Andersen G., Knight R. (2009). PyNAST: A flexible tool for aligning sequences to a template alignment. Bioinformatics.

[203] Wang Q., Garrity G.M., Tiedje J.M., Cole J.R. (2007). Naive Bayesian classifier for rapid assignment of rRNA sequences into the new bacterial taxonomy. Appl. Environ. Microbiol..

[204] Kwak M.-J., Kong H.G., Choi K., Kwon S.-K., Song J.Y., Lee J., Lee P.A., Choi S.Y., Seo M., Lee H.J. (2018). Rhizosphere microbiome structure alters to enable wilt resistance in tomato. Nat. Biotechnol..

[205] Zhang J., Liu Y.-X., Zhang N., Hu B., Jin T., Xu H., Qin Y., Yan P., Zhang X., Guo X. (2019). NRT1.1B is associated with root microbiota composition and nitrogen use in field-grown rice. Nat. Biotechnol..

[206] Edgar R.C. (2013). UPARSE: Highly accurate OTU sequences from microbial amplicon reads. Nat. Methods.

[207] Rideout J.R., He Y., Navas-Molina J.A., Walters W.A., Ursell L.K., Gibbons S.M., Chase J., McDonald D., Gonzalez A., Robbins-Pianka A. (2014). Subsampled open-reference clustering creates consistent, comprehensive OTU definitions and scales to billions of sequences. PeerJ.

[208] Edgar R.C.J.B. (2018). Updating the 97% identity threshold for 16S ribosomal RNA OTUs. Bioinformatics.

[209] Louca S., Parfrey L.W., Doebeli M. (2016). Decoupling function and taxonomy in the global ocean microbiome. Science.

[210] Omojate G.C., Enwa F.O., Jewo A.O., Eze C.O. (2014). Mechanisms of antimicrobial actions of phytochemicals against enteric pathogens—A review. J. Pharm. Chem. Biol. Sci..

[211] Pimentel M.R., Molina G., Dionísio A.P., Junior M.R.M., Pastore G.M. (2011). The Use of Endophytes to Obtain Bioactive Compounds and Their Application in Biotransformation Process. Biotechnol. Res. Int..

[212] Shukla S., Habbu P., Kulkarni V., Jagadish K., Pandey A., Sutariya V. (2014). Endophytic microbes: A novel source for biologically/pharmacologically active secondary metabolites. Asian J. Pharm. Toxicol..

[213] Strobel G.A. (2003). Endophytes as sources of bioactive products. Microbes Infect..

[214] Liarzi O., Bucki P., Miyara S.B., Ezra D. (2016). Bioactive Volatiles from an Endophytic Daldinia cf. concentrica Isolate Affect the Viability of the Plant Parasitic Nematode Meloidogyne javanica. PLoS ONE.

[215] Nongkhlaw F.M., Joshi S.R. (2015). Investigation on the bioactivity of culturable endophytic and epiphytic bacteria associated with ethnomedicinal plants. J. Infect. Dev. Ctries..

[216] Gouda S., Das G., Sen S.K., Shin H.-S., Patra J.K. (2016). Endophytes: A treasure house of bioactive compounds of medicinal importance. Front. Microbiol..

