# Supplementary material for: Diversity and Taxonomic Distribution of Endophytic Bacterial Community in the Rice Plant and Its Prospective
Source: Int J Mol Sci. 2021 Sep 21;22(18):10165. doi: 10.3390/ijms221810165 (PMC8465699; doi:10.3390/ijms221810165)
Supplement: Supplementary file 1 [file ijms-22-10165-s001.zip › ijms-1350558-supplementary.pdf]

International Journal of *Molecular Sciences*

MDPI

Supplementary Materials

Diversity and Taxonomic Distribution of Endophytic Bacterial Community in the Rice Plant and Its Prospective

Mohsin Ali [1,2], Qurban Ali [3,*], Muhammad Aamir Sohail [4], Muhammad Furqan Ashraf [5], Muhammad Hamzah Saleem [6], Saddam Hussain [7] and Lei Zhou [1,*]

1 State Key Laboratory for Managing Biotic and Chemical Threats to the Quality and Safety of Agro-Products, Institute of Agro-Product Safety and Nutrition, Zhejiang Academy of Agricultural Sciences, Hangzhou 310021, China; moh.uaf2356@outlook.com
2 State Key Laboratory of Microbial Resources, Institute of Microbiology, Chinese Academy of Sciences, Beijing 100101, China
3 Key Laboratory of Integrated Management of Crop Diseases and Pests, Ministry of Education, Department of Plant Pathology, College of Plant Protection, Nanjing Agricultural University, Nanjing 210095, China
4 Center for Excellence in Molecular Plant Sciences, National Key Laboratory of Plant Molecular Genetics, Institute of Plant Physiology and Ecology, Chinese Academy of Sciences, 300 Fenglin Road, Shanghai 200032, China; amirsohail306@gmail.com
5 College of Life Sciences, South China Agricultural University, Guangzhou, 510642, China; furqan2210uaf@hotmail.com
6 College of Plant Science and Technology, Huazhong Agricultural University, Wuhan 430070, China; saleemhamza312@webmail.hzau.edu.cn
7 Department of Agronomy, University of Agriculture, Faisalabad 38040, Punjab, Pakistan; sadamhussainuaf@gmail.com
* Correspondence: qurbanalirattar@webmail.hzau.edu.cn (Q.A.); zhoul@zaas.ac.cn (L.Z.)

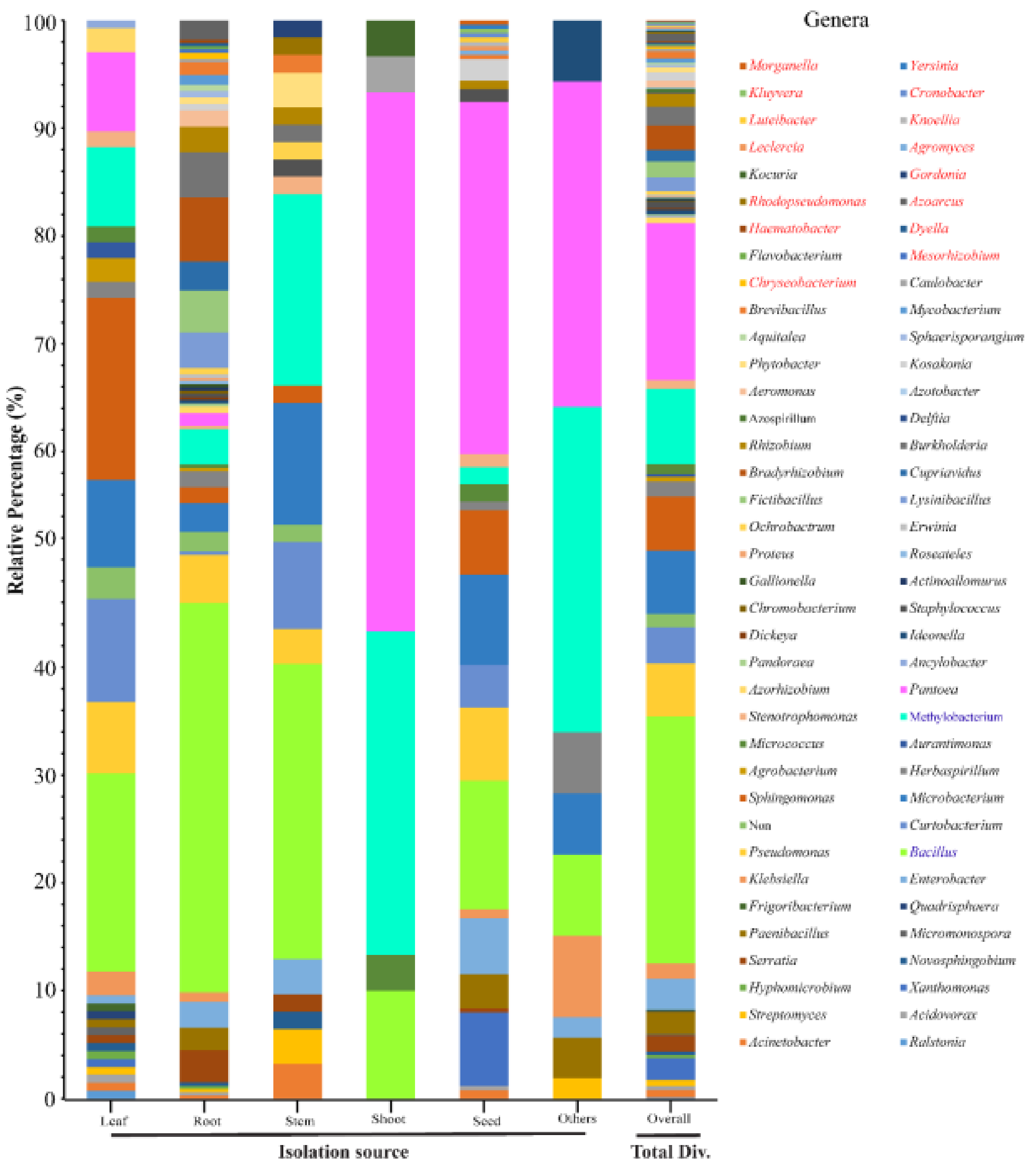

Figure S1. Relative abundance of rice endophytic bacterial diversity at genera level within rice tissues, the isolation sources were presented on the horizontal axis and relative abundance of genera is visualized by the vertical axis in percentage (%), each genus codded with specified color in stacked columns, in legends fifteen genera with red color illustrated that these genera only were detected within same isolation source whereas two genera (*Bacillus and Methylobacterium*) overlapped within isolation sources, Total Div.: presenting the total diversity within rice tissues (Leaf, Root, Stem, Shoot, Seed and including other sources), Other: including isolation sources of rice (Grain filling, Tillage stages and also not-conformed sources).
